# A new strategy for exploring the hierarchical structure of cancers by adaptively partitioning functional modules from gene expression network

**DOI:** 10.1038/srep28720

**Published:** 2016-06-28

**Authors:** Junmei Xu, Runyu Jing, Yuan Liu, Yongcheng Dong, Zhining Wen, Menglong Li

**Affiliations:** 1 College of Chemistry, Sichuan University, Chengdu, China; 2 College of Life Sciences, Sichuan University, Chengdu, China

## Abstract

The interactions among the genes within a disease are helpful for better understanding the hierarchical structure of the complex biological system of it. Most of the current methodologies need the information of known interactions between genes or proteins to create the network connections. However, these methods meet the limitations in clinical cancer researches because different cancers not only share the common interactions among the genes but also own their specific interactions distinguished from each other. Moreover, it is still difficult to decide the boundaries of the sub-networks. Therefore, we proposed a strategy to construct a gene network by using the sparse inverse covariance matrix of gene expression data, and divide it into a series of functional modules by an adaptive partition algorithm. The strategy was validated by using the microarray data of three cancers and the RNA-sequencing data of glioblastoma. The different modules in the network exhibited specific functions in cancers progression. Moreover, based on the gene expression profiles in the modules, the risk of death was well predicted in the clustering analysis and the binary classification, indicating that our strategy can be benefit for investigating the cancer mechanisms and promoting the clinical applications of network-based methodologies in cancer researches.

Researches on analyzing the functions of individual gene have contributed to finding potential oncogenes involved in the initiation and progression of tumors that may be potential targets of anticancer drugs and can be applied to the prognosis of patients^1^,^2^,^3^,^4^,^5^,^6^,^7^,^8^. Although the biological studies of individual gene play a vital role in clinical treatment, the emergent functions and behaviors of individual genes in the context of the whole biological systems still require further investigation^9^,^10^. As the biological function is usually completed by the cooperation of a set of genes, the gene network is one of the suitable tools for better understanding the molecular mechanisms of biological processes in the whole systems^11^.

The network has been used to represent the interactions or regulations among the elements in the system^12^. The framework of gene network, for which the nodes represent genes and the edges represent the interactions between genes, allows us to explore the integrated functions of the sub-networks as well as the property of the whole network, which serves as a powerful paradigm for genes analysis and provides a system-level understanding of gene functions^13^. Generally, the connections among genes or proteins are created mainly based on the known interactions in the knowledge-based databases, such as Human Protein Reference Database (HPRD)^14^, STRING^15^, and Biomolecular Interaction Network Database (BIND)^16^. A number of algorithms have been developed to partition the whole network into several sub-networks in order that people can investigate the potential relationships among the sub-networks, and subsequently infer the molecular mechanisms of the complex biological systems^17^,^18^,^19^,^20^,^21^,^22^,^23^,^24^,^25^,^26^,^27^,^28^,^29^,^30^. Recently, Ghiassian *et al*. successfully extracted the disease modules by systematically analyzing the protein-protein interaction networks of 70 diseases and discovered the pathobiological relationships among these diseases with the identified modules^31^. Menche *et al*. further elucidated the pathobiological relationships among the diseases based on the similarities of the disease modules^32^.

In clinical cancer researches, the gene networks are also benefit for exploring the hierarchical structure of the biological system of a complex cancer^33^ and identifying the functional gene sets that are highly associated with the clinical outcomes. However, the networks constructed with the knowledge-based interactions cannot reflect the heterogeneity of the cancer outcomes very well. Therefore, considering that the gene expression profiling measured by microarray or RNA-sequencing technology is one of the common elements for investigating the molecular mechanisms of cancers, it is essential to develop a strategy, which can construct the gene network only with the gene expression data instead of the known interactions in the database.

In our study, we presented a strategy to generate the potential connections among the genes in a specific cancer with the gene expression profiles, and adaptively divide the whole network into several functional modules, in which the genes directly connected with each other shared similar biological functions. Our proposed strategy was validated by using the microarray data of acute myeloid leukemia (AML), glioblastoma and neuroblastoma, as well as the RNA-sequencing data of glioblastoma (glioblastoma_seq). The gene set enrichment analysis for the genes in the functional modules clearly showed the similarities and differences among the three cancers. Kaplan-Meier analysis showed that the patients with diverse survival durations were well discriminated based on the expression profiles of the genes in functional modules, indicating that our strategy can successfully generate the networks with the gene expression data and extract the functional modules that are highly associated with cancer outcomes. Furthermore, the binary classification with the re-sampling method showed the robust performance of the predictive models on predicting the clinical outcomes of cancers.

## Results

### The gene networks constructed with the gene expression data displayed the scale-free characteristics

In our study, gene networks of three cancers were constructed by using graphical lasso based on gene expression profiles. All of them followed the power-law degree distribution, which were depicted in Supplementary Figs S1–S4. The networks were scale-free networks, in which most of the genes had a few neighborhoods, but some of them had many directly connected genes^34^.

### The top 20 genes ranked by degrees in the networks that were used for generating the seed modules

The top 20 hubs (genes) with high degrees were separately selected from the gene networks of AML, glioblastoma and neuroblastoma. The gene functions were searched in Tumorportal (http://www.tumorportal.org/) and GeneCards (http://www.genecards.org/) to obtain the gene functions.

Among the top 20 genes in AML gene network, four genes had been reported that were correlated with AML. The detailed information about the 20 genes was shown in Supplementary Table S1.

For the hubs in the glioblastoma gene network, ten genes had been reported to be associated with glioblastoma in previous researches. Details of these genes were listed in Supplementary Table S2.

As to the neuroblastoma gene network, the degrees of *NTRK1* and *CDH19* were much larger than those of other genes. Five out of 20 genes were directly related with neuroblastoma (Supplementary Table S3).

For the 20 hubs in the RNA-sequencing data of glioblastoma network, seven genes had reported to be related with glioblastoma (Supplementary Table S4).

### Functional modules derived from the gene networks were associated with specific functions

In this study, two functional modules were separately generated for each gene network. We conducted Gene Ontology (GO) functional annotation and pathway analysis with the genes in each of the modules by using Database for Annotation, Visualization, and Integrated Discovery (DAVID) Functional Annotation Chart tool^35^ and found the diverse functions between the modules of three cancers (Supplementary Tables S5–S12). For each gene network, the module related with the cancer progression was marked as module I, while another one was marked as module II.

### The expression profiles of the genes in functional modules were correlated with the survival outcomes of patients

**Figure 1:**
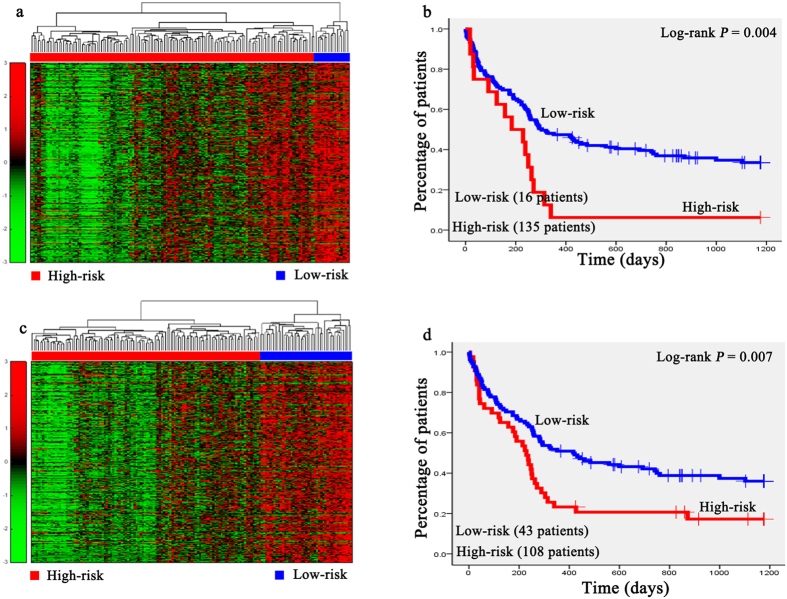
Unsupervised hierarchical clustering of genes in two modules and the Kaplan-Meier analysis of 151 patients with AML. (**a**,**c)** Unsupervised hierarchical clustering of tumor growth module and substance biosynthetic and metabolic module of AML. According to expression profiles of 1484 genes and 738 genes in two modules, 151 patients were clustered into two groups. The blue bar above the patients indicates the low risk group, the red bar indicates the patients with high risk. (**b**,**d**) Kaplan-Meier survival plots of low risk and high risk AML patients based on the hierarchical clustering results of (**a**,**b**) The gene expression signature of the patients in high risk (red lines) with a significantly increased risk of death when compared to low risk group (blue lines).

**Figure 2:**
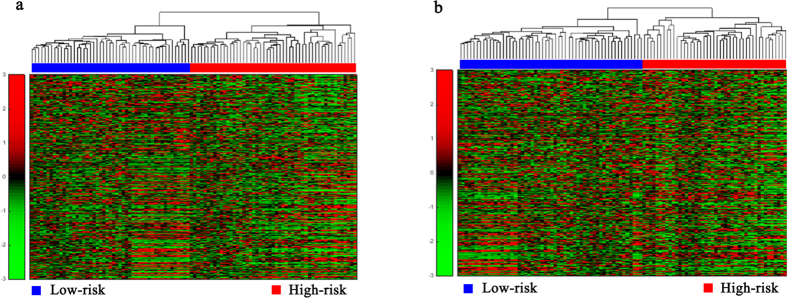
Unsupervised hierarchical clustering of genes in two modules of 100 patients with glioblastoma. (**a**,**b**) Unsupervised hierarchical clustering of the tumor proliferation and progression modules and cellular signaling transduction module of glioblastoma. According to expression profiles of 3710 genes and 1875 genes in two modules, 100 patients were clustered into two groups. The blue bar above the patients indicates the patients belong to the low risk group, the red bar indicates the patients with high risk.

**Figure 3:**
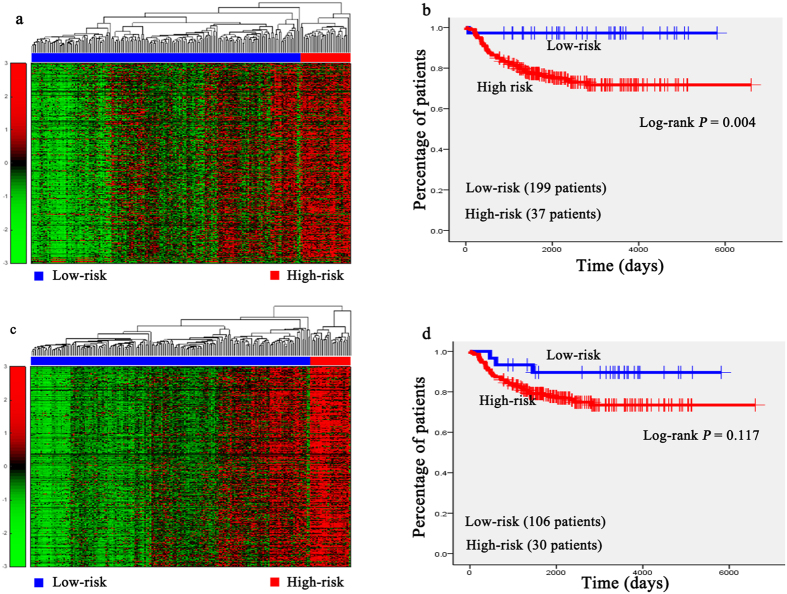
Unsupervised hierarchical clustering of genes in two modules and the Kaplan-Meier analysis of 236 patients with neuroblastoma. (**a**,**c**) Unsupervised hierarchical clustering of apoptosis and tumors migration module and nervous system development module of neuroblastoma. According to expression profiles of 3334 genes and 1739 genes in two modules, 236 patients were clustered into two groups. The blue bar above the patients indicates the low risk group, the red bar indicates the patients with high risk. (**b**,**d**) Kaplan-Meier survival plots of low risk and high risk neuroblastoma patients based on the hierarchical clustering results of (**a**,**b**) The gene expression signature in apoptosis and tumors migration module of the patients in high risk (red lines) with a significantly increased risk of death when compared to low risk group (blue lines).

**Figure 4:**
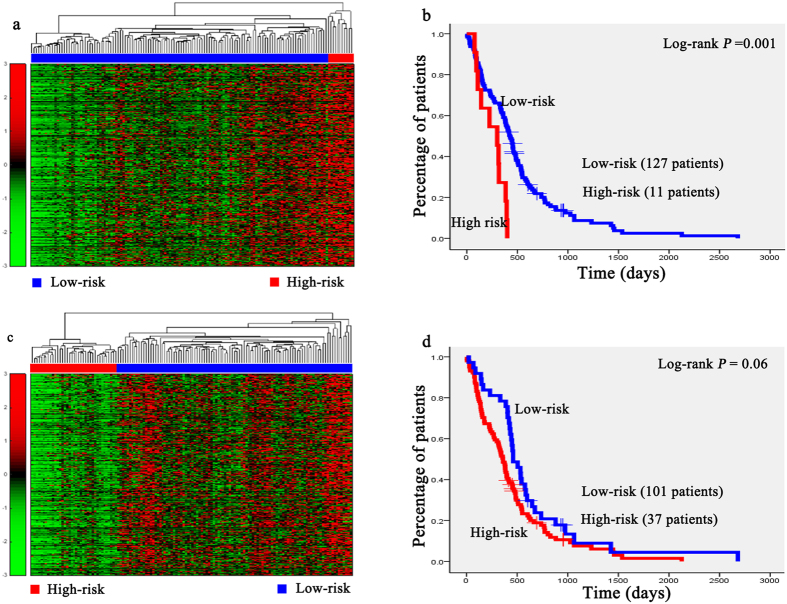
Unsupervised hierarchical clustering of genes in two modules and the Kaplan-Meier analysis of 138 glioblastoma patients of the RNA-sequencing dataset. (**a**,**c**) Unsupervised hierarchical clustering of apoptosis and cell death regulation module and substance biosynthesis and metabolism module of RNA-sequencing data of glioblastoma. According to expression profiles of 1417 genes and 794 genes in two modules, 138 patients were clustered into two groups. The blue bar above the patients indicates the low risk group, the red bar indicates the patients with high risk. (**b**,**d**) Kaplan-Meier survival plots of low risk and high risk RNA-sequencing data of glioblastoma patients based on the hierarchical clustering results of (**a**,**b**). The gene expression signature in apoptosis and cell death regulation module of the patients in high risk (red lines) with a significantly increased risk of death when compared to low risk group (blue lines).

**Figure 5:**
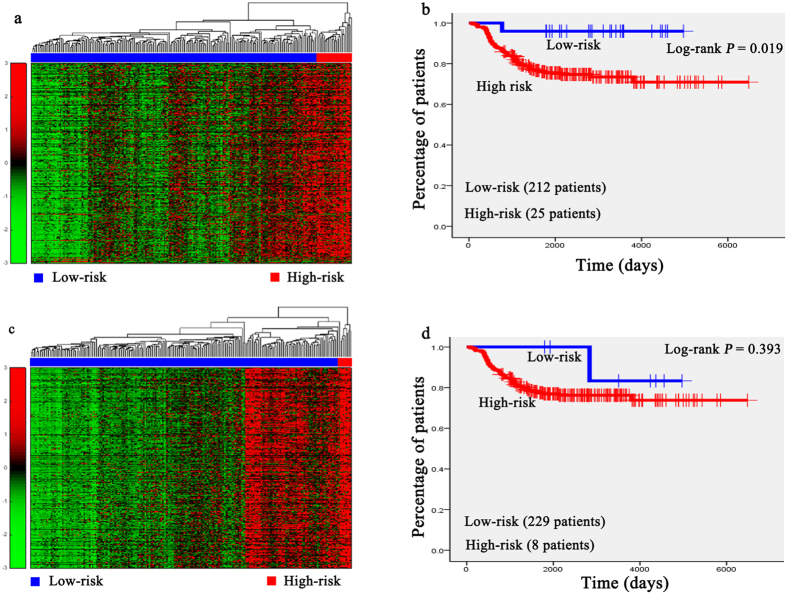
Unsupervised hierarchical clustering of genes in two modules and the Kaplan-Meier analysis of the independent testing set of 237 neuroblastoma patients. (**a**,**c**) Unsupervised hierarchical clustering of two modules of independent testing set. According to expression profiles of 3334 genes and 1739 genes in two modules, 237 patients were clustered into two groups. The blue bar above the patients indicates the low risk group, the red bar indicates the patients with high risk. (**b**,**d**) Kaplan-Meier survival plots of low risk and high risk neuroblastoma patients based on the hierarchical clustering results of (**a**,**b**). The gene expression signature in module I of the patients in high risk (red lines) with a significantly increased risk of death when compared to low risk group (blue lines).

Hierarchical clustering analysis was conducted with the gene expression profiles as features to classify the patients. The 151 AML patients, 100 glioblastoma patients, 236 neuroblastoma patients, 138 RNA-sequencing data of glioblastoma patients, and the 237 independent testing set of neuroblastoma patients were separately grouped into two classes via hierarchical clustering (see Figs 1a,c, 2a,b, 3a,c, 4a,c and 5a,c).

Kaplan-Meier survival analysis was subsequently performed on the patients with AML, neuroblastoma, glioblastona_seq, and independent testing set of neuroblastoma (Figs 1b,d, 3b,d, 4b,d, 5b,d). The results suggested that the patients in AML, neuroblastoma, RNA-sequencing data of glioblastoma, and independent testing set of neuroblastoma datasets with shorter survival durations can be well discriminated by using the expression profiles of the genes in cancer-related functional modules.

### Binary classification with the genes in functional modules

**Figure 6:**
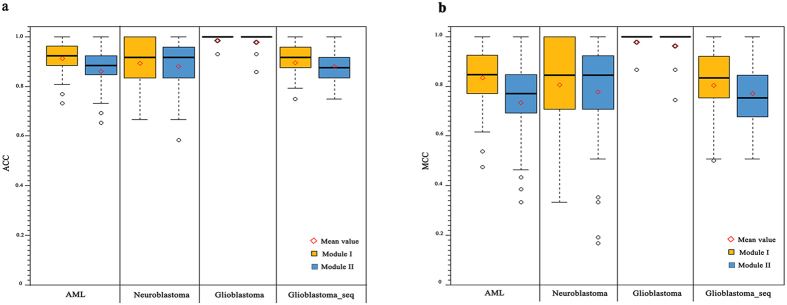
Performance of the 100 times re-sampling for each module of four datasets. (**a**) The distribution of ACC values of the 100 times re-sampling for module I (left, yellow) and module II (right, blue) of four datasets. (**b**) The distribution of MCC values of the 100 times re-sampling for module I (left, yellow) and module II (right, blue) of four datasets. is the mean value of the 100 times random re-sampling of each module.

The averaged performance of the 100 times random re-sampling of the AML, glioblastoma, neuroblastoma, and the RNA-sequencing data of glioblastoma datasets was shown in Table 1. According to Table 1, all the features derived from module I achieved better performance than that derived from the module II, and the averaged ACCs for the module I of AML, glioblastoma, neuroblastoma, and RNA-sequencing data of glioblastoma were 0.919, 0.991, 0.899, and 0.902. The averaged MCCs for the functional modules of four datasets and the distribution for 100 times random re-sampling with the ACC and MCC were shown in Fig. 6. The performance of the independent testing set of neuroblastoma was shown in Table 2.

## Discussion

In this study, we proposed a strategy for gene network analysis, which is based on the gene expression data and can adaptively divide the whole network into several functional modules. The microarray data sets of three cancers, namely AML, glioblastoma and neuroblastoma, as well as the RNA-sequencing dataset of glioblastoma, were applied to evaluate the performance of our strategy. Based on the gene expression profiles of the patients, we separately constructed the gene networks for four datasets and obtained two functional modules from each of the networks. In general, one functional module was related to the tumor initiation and progression (module I), and another one was mainly associated with the cellular process (module II). According to the hierarchical clustering and Kaplan-Meier analysis, the gene expression profiles in module I achieved better discrimination of the patients’ risk group. To tested whether the clustering results achieved with the genes in functional modules are better than those obtained with the randomly selected genes by using the Monte Carlo sampling procedure. In total, 1484, 3710 and 3334 genes were grouped into the cancer related modules for the datasets of AML, glioblastoma and neuroblastoma, respectively, and used in the subsequent clustering analysis and survival analysis. So, for each of the datasets, we randomly selected the same number of genes from the gene network and used them for clustering analysis and survival analysis. This procedure has been repeated for 100 times and the p values obtained by log-rank test (conducted in survival analysis) were shown in Supplementary Fig. S5. From the figure, we find that the p values (p = 0.004, 0.004, and 0.001 for AML, neuroblastoma, and glioblastoma, resp.) obtained by using the genes in module I were less than those (average p value = 0.057, 0.125, and 0.160 for AML, neuroblastoma, and glioblastoma, resp.) obtained in the 100 re-sampling procedures. We also used all the differentially expressed genes (DEGs) for the clustering analysis and the survival analysis, the log-rank p values were 0.004, 0.008, and 0.211 for AML, neuroblastoma, and glioblastoma, respectively. We considered that, to some extent, the genes in the functional modules performed better than those selected by chance in clustering the patients into high/low risk groups.

**Figure 7:**
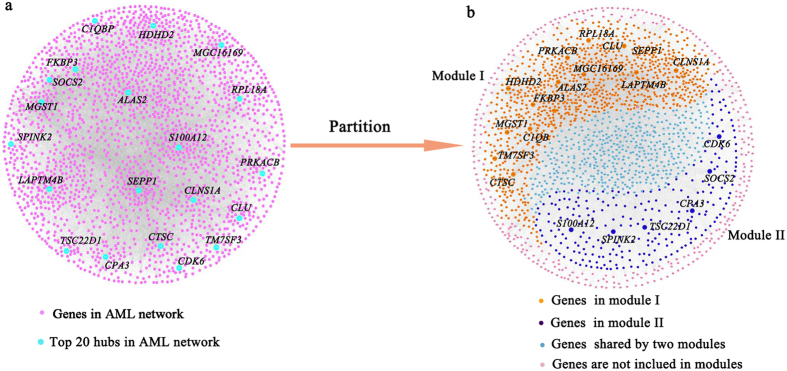
Network and the functional modules of AML. (**a**) The visualization of AML gene network. (**b**) The visualization of the functional modules in the gene network.

The gene networks were constructed by using graphical lasso based on gene expression profiles. In the AML gene networks, the degrees of a few genes were very larger than the most genes in the network, which were shown in Fig. 7. The node degree distribution of the AML gene network followed a power-law distribution (Supplementary Fig. S1), which was a characteristic of scale-free networks^36^. The other three gene networks also showed the same property (Supplementary Figs S2–S4). The top 20 genes ranked by degrees and their first neighbors in the networks were the basis of our strategy to identify the functional modules. Thus, the scale-free topology of the gene networks was necessary in this study.

In the gene network of AML, *SOCS2* and *CDK6* were involved in two modules. The overexpression of *SOCS2* in AML patients indicates the favorable prognosis^37^. *CDK6* plays an vital role in mixed-lineage leukemia fusions in myeloid leukemogenesis, which is associated with poor prognosis of patients^38^. In the module related to the tumor growth (Supplementary Table S5), the GO terms of damaged DNA binding was significantly enriched (*p* = 2.09 × 10^−2^), which promoted to tumor suppression^39^. The enriched biological process (BP) of glycolysis (*p* = 4.7 × 10^−2^) is usually active in tumor cells and the intermediates were involved in tumor tissues^40^. The oxidative phosphorylation pathway (*p* = 1.59 × 10^−9^) was significantly enriched in tumor growth module, which decreased the availability of ATP that associated with malignancies and tumor cell expansion and was reported to be one of the key progress in tumor growth^41^. In particular, the level of oxidative phosphorylation pathway might leads to the changes of mitochondrial functions, which was the character of the hematological cell malignancies, AML was included^42^. In the module related to the biosynthesis and metabolism of substance, the pathways of steroid biosynthesis (*p* = 3.50 × 10^−3^), pyruvate metabolism (*p* = 1.81 × 10^−2^), and amino sugar and nucleotide sugar metabolism (*p* = 2.64 × 10^−2^) were significantly enriched, which mainly contributed to the biosynthetic and metabolic processes of the proteins, steroids and pyruvate. The GO terms and pathways enriched with the genes in this module were listed in Supplementary Table S6. From the results of Kaplan-Meier survival analysis (Fig. 1b,d), we found that the difference of survival durations of the patients between high- and low-risk groups separated by the gene expression profiles in the module related to tumor growth was more significant than that separated by the gene expression profiles in another module, indicating that the genes involved in the initiation and progression of AML were more suitable for the prognosis of patients.

For glioblastoma, in the module of tumor proliferation and progression, the hub *FSTL5*, as a candidate gene for tumor-suppressor, encodes a secretory glycoprotein and its expression level is highly correlated with tumor size. *IL13RA2* is a tumor antigen-like factor, which is highly expressed in the glioblastoma patients^43^. Two enriched GO terms named cell morphogenesis involved in differentiation (*p* = 1.24 × 10^−5^) and cell migration (*p* = 2.59 × 10^−6^) mainly contribute to the tumor progression and migration. MAPK signaling pathway (*p* = 4.31 × 10^−4^) and the pathway in cancer (*p* = 3.48 × 10^−3^) were also significantly enriched in this module (Supplementary Table S7). The enriched Wnt signaling pathway (*p* = 4.73 × 10^−5^) had been reported in previous study that it might contribute to glioma cell proliferation and impaired apoptosis, which allowed for the progression to glioblastoma^44^. Genes in axon guidance pathway (*p* = 3.39 × 10^−3^) were reported to be differentially expressed in the patients with Parkinson disease compared to the healthy ones, which are the signs of the occurrence of the brain disorder^45^. In the module about cellular signaling transduction, the enriched GO terms named intracellular signaling cascade (*p* = 3.60 × 10^−7^), regulation of cell communication (*p* = 1.12 × 10^−5^) and ion transport (*p* = 1.30 × 10^−5^) contribute to the transduction among the cells. The most significantly enriched calcium signaling pathway (*p* = 1.82 × 10^−4^) in this module, was involved in regulating neural functions such as brain rhythms generation and information transduction in the processes of memory and learning^46^. The pathways about cellular signaling transduction were also significantly enriched with the genes in this module (Supplementary Table S8).

In the gene network of neuroblastoma, one functional module was about cell apoptosis and tumor migration and another was about the development of nervous system. The hub *NTRK1* was involved in two functional modules. It is high expressed in the low-risk group of neuroblastoma patients, indicating the favorable prognosis of neuroblastoma patients^47^. In the module about cell apoptosis and tumor migration, Pathway of apoptosis was a genetically controlled mechanism of cell death involved in the regulation of tissue homeostasis. MAPK signaling pathway (*p* = 4.23 × 10^−3^) was activated in human cancers and resulted in malignant phenotypes^48^. The pathway of cell adhesion molecules (CAMs) (*p* = 2.27 × 10^−8^) was involved in tumors migration^49^. All the enriched GO terms and pathways for this module were listed in Supplementary Table S9. In the module about the development of nervous system, the enriched GO terms of the ectoderm development (*p* = 8.88 × 10^−6^) and organ development (*p* = 1.26 × 10^−8^) are the final steps in the development of nervous system. The pathway of neuroactive ligand-receptor interaction (*p* = 1.11 × 10^−5^) was associated with the neuronal function^50^. The pathway of cytokine–cytokine interactions (*p* = 1.08 × 10^−3^) is crucial during immunological and inflammatory responses in disease^51^. The detailed results of the DAVID analysis for this module were listed in Supplementary Table S10. Based on the expression profiles of the genes in these two modules, the patients were significantly divided into high-risk and low-risk groups by hierarchical clustering analysis (Fig. 3b,d). Note that the difference of survival durations of the patients between high- and low-risk groups separated by the gene expression profiles in the module related to cell apoptosis and tumor migration was more significant than that separated by the gene expression profiles in another module.

For the gene network constructed based on RNA-sequencing data of glioblastoma, one of the functional modules was mainly about apoptosis and programmed cell death regulation, and the other one was about substance biosynthesis and metabolism. Expression of *PI3* could be induced by inflammatory mediators such as tumor necrosisfactor and the interleukin 1 beta^52^. The *L1CAM* could stimulate glioblastoma cells motility and proliferation^53^. In the module about apoptosis and programmed cell death regulation, GO terms enriched in it were tumor necrosis factor (TNF) binding (*p* = 2.71 × 10^−2^), tumor necrosis factor receptor superfamily binding (*p* = 2.90 × 10^−2^). TNF has been reported involved in the cancers development and progression in preclinical models^54^. The pathways enriched in this modules were cytokine–cytokine interactions (*p* = 4.06 × 10^−12^), apoptosis (*p* = 1.34 × 10^−3^) (Supplementary Table S11). In the module of substance biosynthesis and metabolism, the enriched GO terms were sulfuric ester hydrolase activity (*p* = 6.83 × 10^−5^), cytokine activity (*p* = 2.07 × 10^−4^), the enriched pathway were ECM-receptor interaction(*p* = 3.86 × 10^−5^), keratan sulfate biosynthesis (*p* = 3.59 × 10^−3^), and fructose and mannose metabolism (*p* = 2.20 × 10^−2^). The results of DAVID analysis were shown in Supplementary Table S12. Fig. 4b,d showed that the difference of survival durations of the patients between high- and low-risk groups separated by the gene expression profiles in the module related to apoptosis and programmed cell death regulation was more significant than that separated by the gene expression profiles in another module, indicating that the genes involved in the cancers progression were more suitable for the prognosis of patients. Our strategy could be applied to both microarray and RNA-sequencing data.

We further investigated the differences and similarities of the modules among three microarray datasets. The overlaps of the genes in the modules that related to the cancer initiation and progression and cellular process were shown in Supplementary Fig. S6. It can be seen that 789 genes (21.3%) in glioblastoma overlapped with neuroblastoma in cancer initiation and progression (Supplementary Fig. S6a), while 353 genes (9.5%) in glioblastoma overlapped with AML. Likewise, 109 genes (5.8%) in glioblastoma overlapped with neuroblastoma in cellular process (Supplementary Fig. S6b), while 79 genes (4.2%) in glioblastoma overlapped with AML. It indicates that the molecular basis of glioblastoma was similar to that of neuroblastoma.

Furthermore, we evaluated the genes predictive ability for the patients’ cohort in the functional modules by using SVM modeling. To avoid the overfitting, feature selection was applied to reduce dimension of the genes for each dataset. As the models are often not robust, re-sampling is a good manner to test the robustness of the models, and Li *et al*. achieved the high quality cancer signature genes identification by MSS algorithm with re-sampling test^55^,^56^. According to averaged values of the evaluation parameters in Table 1, the features selected from functional modules achieved a good performance for predicting the patients risk group. The ACC and MCC distribution of 100 times re-sampling (Fig. 6) suggested that our models were robust to predict the patients’ cohorts.

In summary, our strategy can generate the gene network based on the gene expression data and adaptively divide the whole network into the functional modules. To facilitate the clinical application, the gene network can be constructed only with the gene expression data by using our proposed strategy. Considering the limited information of gene expression data, not all the genes in the functional modules are necessarily associated with cancers. Therefore, before using these genes for the subsequent analysis and prediction, it is still necessary to refine the genes by using the knowledge-based datasets, such as the curated human signaling network (http://www.cancer-systemsbiology.org/dataandsoftware.htm), which is created by manually collecting the data sources from multiple pathway-related databases (signaling network of BioCarta, CST Signaling pathways, Pathway Interaction, iHOP) and literatures, and successfully applied in the identification of cancer biomarkers^55^,^57^,^58^,^59^. In this study, the two modules extracted from the gene networks of three cancers exhibited distinct functions, which were associated with the cancer initiation and progression, and cellular process, respectively. The gene expression profiles in both two modules were significantly related with the pathogenesis and prognosis of these cancers, and can well discriminate the patients with shorter survival durations from the cancer patient cohort.

## Material and Methods

### Datasets

The microarray datasets of AML, glioblastoma and neuroblastoma used in this study were available at the Gene Expression Omnibus (GEO) database and the accession numbers were GSE12417, GSE4271 and GSE49710, respectively. The RNA-sequencing data was downloaded from the Cancer Genome Atlas (TCGA). Neuroblastoma dataset contains 473 samples that are independently profiled with Agilent-020382 Human Custom Microarray 44 k. AML dataset is consist of 151 samples and profiled with Affymetrix microarray HG-U133A&B and HG-133Plus2. Glioblastoma dataset consists of 100 samples and profiled with Affymetrix microarray HG-U133A. The RNA-sequencing data of glioblastoma was generated by Illumina HiSeq RNASeqV2. The raw count for each of genes (data in level 3) was used as expression value. The RNA-sequencing data of glioblastoma dataset consisted of 138 samples. In addition, for the 473 neuroblastoma patients, 236 samples are used to construct gene network, and the others are used as the independent testing set.

For the multiple probesets that mapped to the same HUGO gene symbol, we only kept the probeset with maximum value as the expression level of this gene. The patients in the datasets of AML, neuroblastoma, and the RNA-sequencing data of glioblastoma were divided into two groups according to the survival days. The patients of AML with survival days longer than one year were assigned to the low risk group. Otherwise, patients were assigned to the high risk group. For the neuroblastoma and RNA-sequencing data of glioblastoma datasets, the patients with survival days longer than two years were assigned to the low risk group and the patients with survival days less than two years were assigned to the high risk group. If a patient with the survival days were less than the cut-off days and he/she was still alive, he/she would be removed from the dataset. For the glioblastoma patients, the patients belong to ProNeural (PN) subcategory were assigned to the low risk group and the rest were assigned to the high risk group^60^.

**Figure 8:**
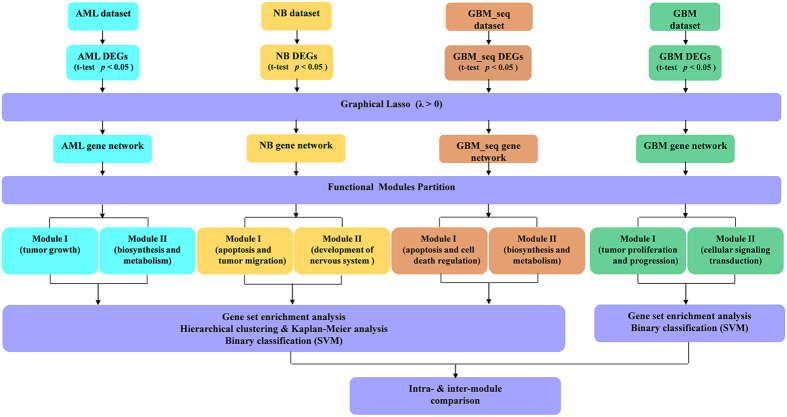
A schematic view of our work in this article as part of the whole research project for functional modules partition. AML: acute myelocytic leukemia. GBM: glioblastoma. NB: neuroblastoma. GBM_seq: RNA-sequencing data set of the glioblastoma. DEGs: differentially expressed genes. SVM: support vector machine.

After the preprocess of the raw four data sets, 14,894, 14,894, 19,860, and 20,531 genes were included in AML, glioblastoma neuroblastoma, and RNA-sequencing data of glioblastoma datasets, too many genes made the construction of gene networks by graphical lasso impossible, thus we need to select a part of genes for following study. Student’s t-test was used to identify the DEGs and those with *p* value < 0.05, and the false discovery rate was not controlled. As a result, 10,184, 2966, 7373 and 2528 DEGs were selected for neuroblastoma, AML, glioblastoma and RNA-sequencing data of glioblastoma, respectively. The overview of this work was shown in Fig. 8.

### Gene-gene network construction

In this work, the most based strategy, i.e. using the inverse of the covariance matrix, was used to build the network. However, the number of DEGs for four datasets are all larger than the number of samples, thus we got a noninvertible covariance matrix^61^. To address this problem, we applied the graphical lasso, an algorithm of estimation of a sparse inverse covariance matrix using L_1_ penalty, to learn the structure in an undirected graphical model^62^. Here, we definite P and N as the number of the gene and sample respectively, ∑ is a covariance matrix which size is P×N.

Let θ = ∑^−1^, and let S be the empirical covariance matrix, the problem is to maximize the penalized log-likehood


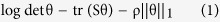


Here tr represents the trace and ρ is a nonnegative tuning parameter; ||θ||_1_ is the L_1_ norm, the sum of the absolute values of the element θ.

The sparse of the θ is decided by L_1_ norm, when ρ is sufficiently large, the estimation of the inverse covariance matrix would be sparse enough, which can help us to find the significant strong interaction among the elements input. While the small of the ρ value means the covariance matrix that we estimated would better explain the model we construct from the data. The topology of the gene networks was estimated by setting the edges to correspond to the nonzero elements of the inverse covariance matrix θ^63^.

In this study, for each dataset, the penalty values for three datasets should meet the needs of modules partition. The penalty parameters were ranking from 0 to 1.0, and the step size was 0.1. The sparsity of the network was proportional to the penalty parameter, the time of computation was increasing with the sparsity of the network as well as the number of elements of the datasets. The penalty parameters for AML, glioblastoma, neuroblastoma and the RNA-sequencing data of glioblastoma were 0.2, 0.7, 1.0 and 0.6, respectively.

### Functional module partition based on the network

For a constructed network (also called graph) G = (V, E), the vertexes (V) and the edges (E) are defined previously, i.e. genes and their relationships. Firstly, we calculated the degree of all the genes and ranked them, the first 20 genes with the largest degree are picked out as the hubs H = {h}_i = 1,…,n_ ∈ V. Then the modules U = {U_i_}_i = 1,…n_ could be initialized based on the hub genes:


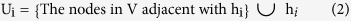


That is, the genes which adjacent with a hub gene are in an initialized module.

If the intersection of two (or more) initialized modules is null, we defined the two (or more) modules as a seed module set. Therefore, the seed modules S = {S_i_}_i = 1,..,m_ could be defined as


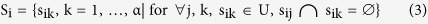


For convenience, we focused on the first seed module set S_1_, the rest modules could be represented as R_1_, i.e.


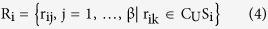


Then we could merge the elements in R_1_ into S_1_ by calculating the overlaps between each r_ij_ and s_ik_:


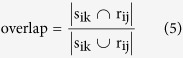


The symbol ‘||’ means the card of a set, i.e. the number of the elements in a set. Every r_ij_ could be merged into a s_ik_ with the largest overlap. Note that after each merging, the merged s_ik_ was updated as the merged set.

After merging, R_1_ was merged into S_1_, the merged set is represented as C_1_. Repeat the steps, all the elements in S could be updated as C = {C_i_}. The elements in C_i_ could be regard as a candidate of the partitioned modules. To find the best partition modules, an evaluation method is needed. In this work, we still evaluated C_i_ = {c_i1_, …, c_i_*α*} by calculating the average overlap:


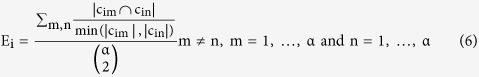



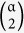
 is the combination number, namely, Binomial coefficient. In our opinion, if two modules are well divided, the average overlap of the modules will be small. Thus, C_i_ with the smallest E_i_ is selected as the best partitioned modules. Moreover, the smallest E_i_ might be not unique (for instance, two C_i_ have the same E_i_), thus the methods might get more than one result based on different seed module sets. However, in this work, even we encounter this situation, that is, two different seed module sets, S_i_ and S_j_, got the same smallest evaluation number but the updated module sets, C_i_ and C_j_, are completely the same.

### Parameter Optimization of the penalty parameter

Note that seed module set (|***S***|≥1) is indispensable, the constructed network would contain at least two initialized modules which have no any common genes. Therefore, the parameter optimization is necessary. Since the limitation of GO analysis, a module which contains more genes could get the enrichment result. Thus our strategy in this work is to get the parameter which could reserve more edge and ensure the constructed network contain at least one seed module set. In this study, the penalty parameters were ranking from 0 to 1.0, and the step size was 0.1. The sparsity of the network was proportional to the penalty parameter, the time of computation was increasing with the sparsity of the network as well as the number of elements of the datasets. The penalty parameters for AML, glioblastoma, neuroblastoma and the RNA-sequencing data of glioblastoma were 0.2, 0.7, 1.0 and 0.6, respectively.

The pseudocodes of section 2 to section 4 are listed in Table 3. Moreover, to provide easy use of this method, the web serve of functional modules partition strategy is available at http://scu-cheminfo.com/GFMP/. The MATLAB code of the strategy and the datasets are also included in this website.

### GO Term Enrichment and Pathway Enrichment Analysis

The Gene Ontology (GO) contains three categories, namely Biological process (BP), Molecular function (MF) and Cellular component (CC), which can consistently describe and annotate gene products^64^. In our study, the enrichment of GO terms was performed by using DAVID (The database for Annotation, Visualization and Integrated Discovery) Functional Annotation Chart tool^35^,^65^ (http://david.abcc.ncifcrf.gov/). The GO terms are organized as a tree structure, wherein term specificity increases and genome coverage decreases as one moves down the tree structure. Level 1 provides the highest list coverage with the least amount of term specificity and level 5 provides the least amount of coverage with the highest term specificity^35^,^66^. Here, in order to avoid very general and uninformative GO terms, we only used the GO terms in level 4. The *p* values were corrected for multiple testing using the Bonferroni procedure and transformed by taking the -log10 for easier visualization^66^.

Based on the DEGs, the pathway enrichment analysis calculates the hypergeometric distribution between the DEGs and pathway and gives a *p* value for each pathway that contains any DEGs. The small *p* value suggests that the DEGs are enriched in the pathway. As DAVID consists of an integrated biological knowledge base and analytic tools aimed at systematically extracting biological meaning from large gene lists, we directly used it to identify the over-represented KEGG pathway^67^. To guarantee the statistically significant in this study, we kept the enriched GO terms and pathways with the *p* value less than or equal to 0.05.

### Feature selection for binary classification

As the number of genes in each functional module is larger than the number of samples in four datasets, overfitting is a challenge when all genes in modules are used for modeling. Thus, the feature selection methods are applied before building modules. 30 genes are selected from every functional module. Then grid search is used for parameter optimization, and the built modules are determined by 5-fold cross-validation. The whole process of the feature selection and modeling are achieved by using a Parallel Machine Learning toolbox for data classification and regression (PML) platform (http://cic.scu.edu.cn/pml/)^68^. Among the thirteen features used selection methods, SVMAttributeEval (An evaluater which select the feature by using an SVM classifier) outperforms other methods. The recursive feature elimination (RFE) evaluates the features by using SVM classifier. Features are assigned a parameter by using the algorithm SVM-train, and then the parameter is used to calculate the weight of the feature by a decision function. RFE is the ranking criterion of the weight of feature. Feature selection is according to the ranks of the features based on the FRE^69^.

### Machine learning with re-sampling procedure

To evaluate the robustness, the re-sampling mechanism is used in this work. SVM is a supervised learning method and often used for binary classification^70^. The random re-sampling was repeated 100 times to select training set and valid set from the AML, neuroblastoma, and RNA-sequencing data of glioblastoma datasets, and the averaged values were used for estimating the robust of the models. The LIBSVM in MATLAB was used for modeling^71^. The training models were determined by grid search and 5-fold cross-validation. The ratio of the positive samples and negative samples was 1:1. The ratio of the training set and validation set was 4:1. Here, sensitivity (SEN), specificity (SPC), accuracy (ACC), and Mathew’s correlation coefficient (MCC) were used as evaluation parameters. The related formulas are as follow:


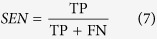



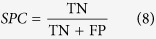



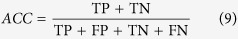



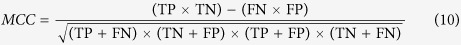


where *TP* (True Positive) is the number of the correctly predicted positive sample, *TN* (True Negative) is the number of the correctly predicted negative sample, *FP* (False Positive) is the number of the incorrectly predicted positive sample, and *FN* (False Negative) is the number of the incorrectly predicted negative sample.

### Hierarchical Clustering survival analysis and network visualization

For the hierarchical clustering analysis, only genes expression profiles in modules were used for clustering. The whole procedure was conducted in MATLAB 8.0. The Kaplan-Meier survival analysis was conducted by using IBM SPSS Statistics 19. The network visualization was conducted by Cytoscape 3.2.0.

### Data availability

 The web serve of functional modules partition strategy is available at http://scu-cheminfo.com/GFMP/. The MATLAB code of the strategy and the datasets are also included in this website. We also upload the codes and the data to GitHub (https://github.com/limlcic/GFMP).

## Additional Information

**How to cite this article**: Xu, J. *et al*. A new strategy for exploring the hierarchical structure of cancers by adaptively partitioning functional modules from gene expression network. *Sci. Rep.* **6**, 28720; doi: 10.1038/srep28720 (2016).

## Supplementary Material

Supplementary Information

## Figures and Tables

**Table 1:** The averaged performance of the 100 times random re-sampling with the SVM models.

	AML		Glioblastoma		Neuroblastoma		Glioblastoma_seq	
	Module I	Module II	Module I	Module II	Module I	Module II	Module I	Module II
SPC	0.925	0.878	0.987	0.967	0.885	0.922	0.888	0.882
SEN	0.912	0.855	0.996	0.999	0.913	0.850	0.915	0.889
ACC	0.919	0.866	0.991	0.983	0.899	0.886	0.902	0.885
MCC	0.841	0.741	0.984	0.968	0.813	0.785	0.810	0.777

**Table 2:** Performance of the independent testing set of the 237 neuroblastoma patients.

Neuroblastoma	SPC	SEN	ACC	MCC
Module I	0.910	0.500	0.865	0.375
Module II	0.886	0.500	0.849	0.333

**Table 3:**
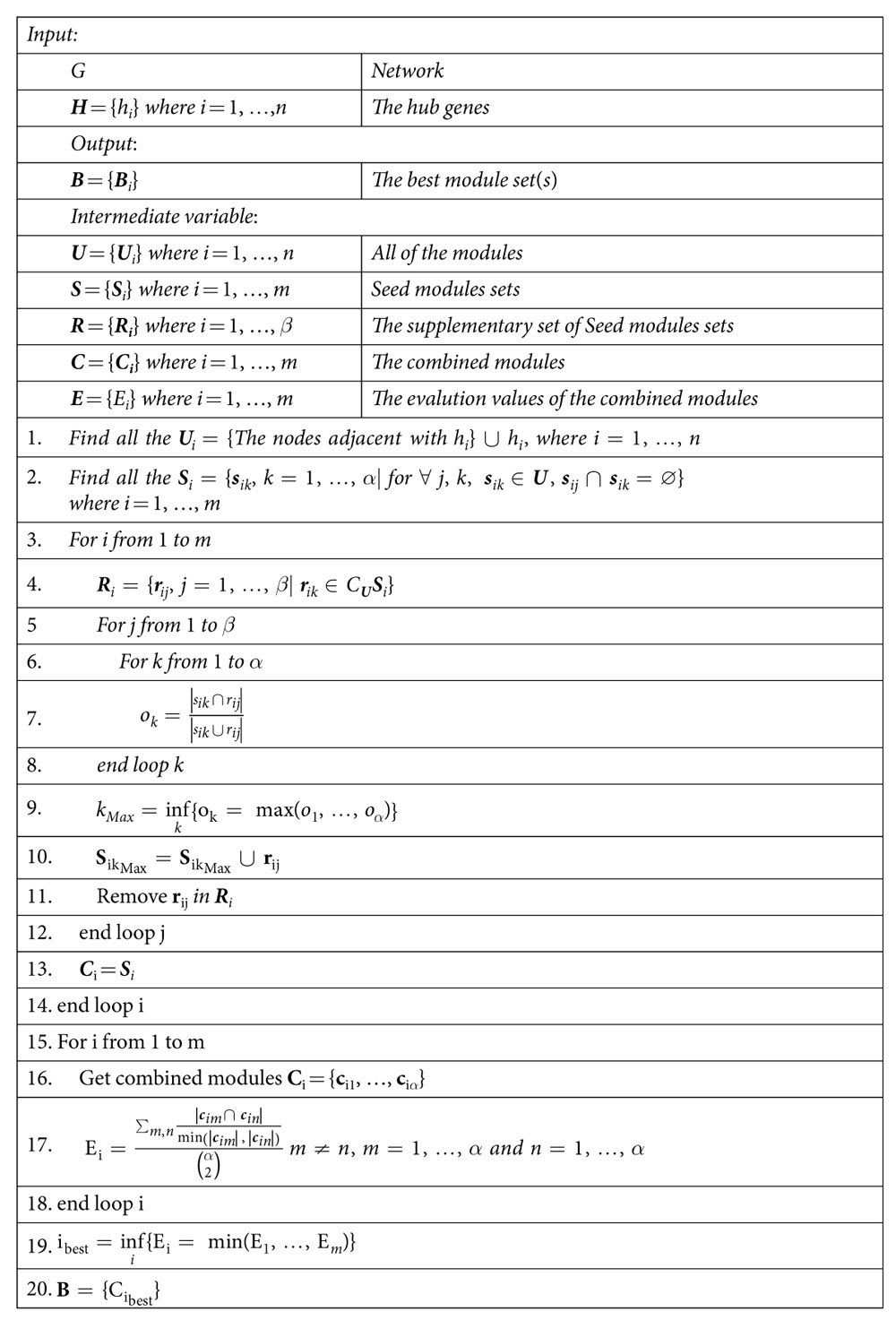
The pseudocode of functional modules partition scheme.

Note: The i_best_ might not unique, Thus the |**B**| could be larger than 1.
