# Supplementary material for: A new strategy for exploring the hierarchical structure of cancers by adaptively partitioning functional modules from gene expression network
Source: Sci Rep. 2016 Jun 28;6:28720. doi: 10.1038/srep28720 (PMC4923884; doi:10.1038/srep28720)
Supplement: Supplementary Information [file srep28720-s1.pdf]

Supplementary Materials for

A new strategy for exploring the hierarchical structure of cancers by adaptively partitioning functional modules from gene expression network

Junmei Xu[1], Runyu Jing[1], Yuan Liu[1], Yongcheng Dong[2], Zhining Wen[1]*, Menglong Li[1]*

Figure S1. Gene degree distribution of the AML gene network
Figure S2. Gene degree distribution of the glioblastoma gene network
Figure S3. Gene degree distribution of the neuroblastoma gene network
Figure S4. Gene degree distribution of the glioblastoma_seq gene network
Figure S5. The log-rank p values of module I and 100 times random re-sampling for AML, neuroblastoma, and glioblastoma.
Figure S6. Genes overlap of the modules of three cancers
Table S1. The top 20 genes ranked by degrees in the AML network that were used for generating the seed modules
Table S2. The top 20 genes ranked by degrees in the glioblastoma network that were used for generating the seed modules
Table S3. The top 20 genes ranked by degrees in the neuroblastoma network that were used for generating the seed modules
Table S4. The top 20 genes ranked by degrees in the glioblastoma_seq network that were used for generating the seed modules
Table S5. Go and Pathway analysis of the tumor cells growth module of AML
Table S6. Go and Pathway analysis of the substance biosynthesis and metabolism module of AML
Table S7. Go and Pathway analysis of the tumor proliferation and progression module of glioblastoma
Table S8. Go and Pathway analysis of the cellular signaling transduction module of glioblastoma
Table S9. Go and Pathway analysis of the apoptosis and tumors migration module of neuroblastoma
Table S10. Go and Pathway analysis of the nervous system development module of neuroblastoma
Table S11. Go and Pathway analysis of the apotosis and cell death regulation module of glioblastoma_seq
Table S12. Go and Pathway analysis of the substance biosynthesis and metabolism module of glioblastoma_seq

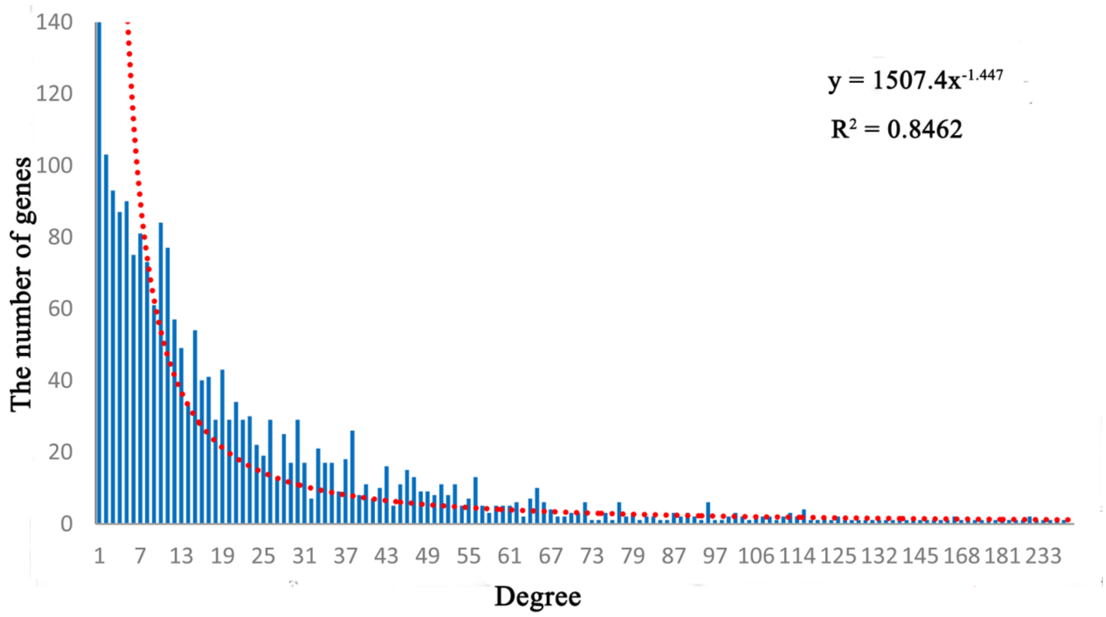

Figure S1. Gene degree distribution of the AML gene network
The blue bars are number of genes in the network, the red line is the fitted curve. R^2 is the coefficient of determination.

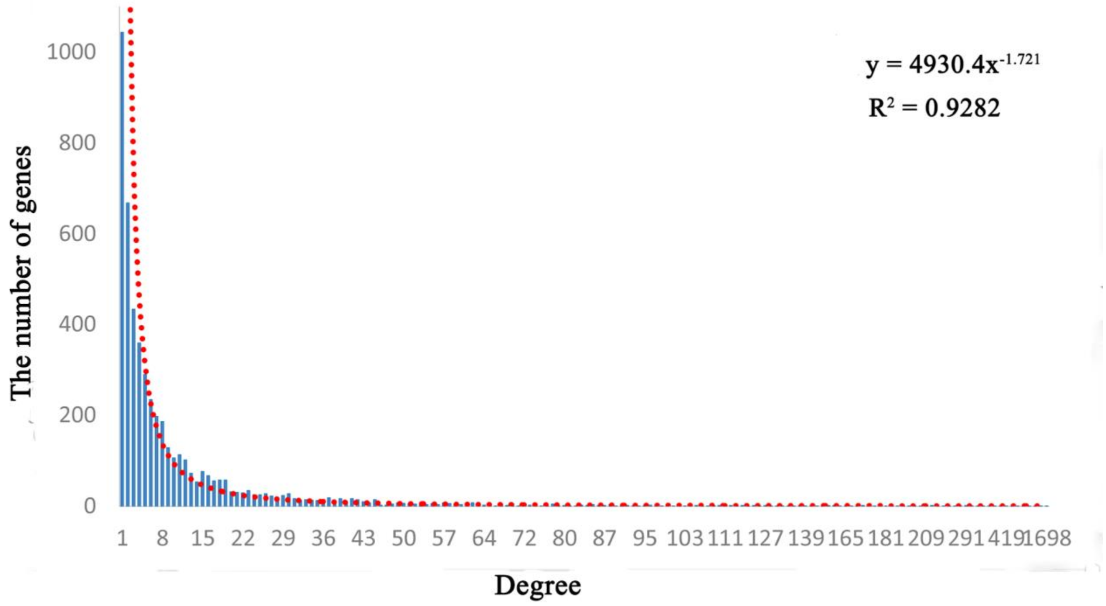

Figure S2. Gene degree distribution of the glioblastoma gene network
The blue bars are number of genes in the network, the red line is the fitted curve. R^2 is the coefficient of determination.

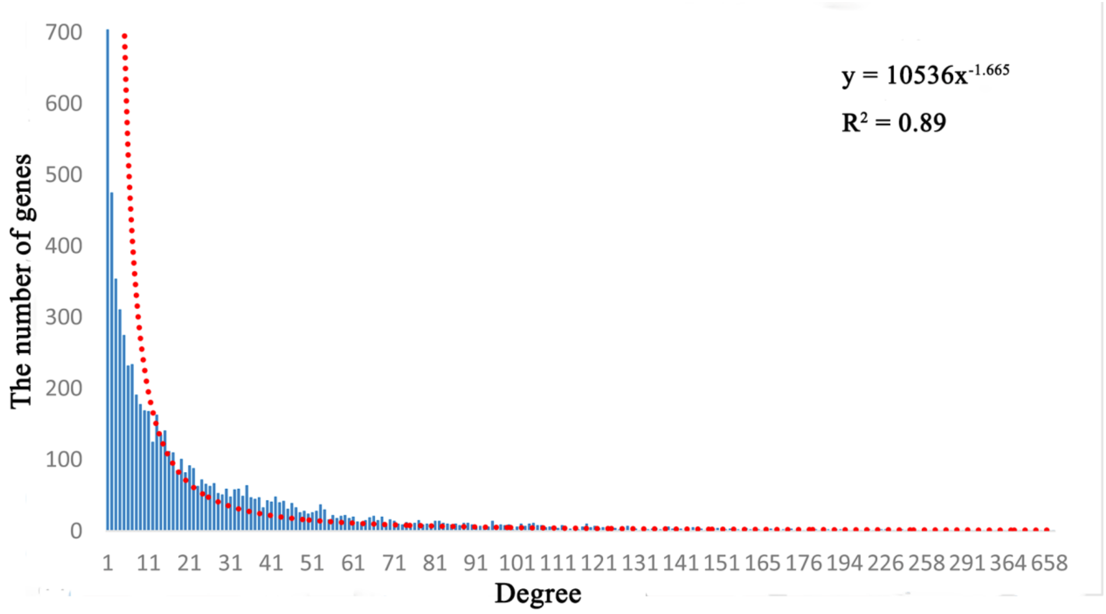

Figure S3. Gene degree distribution of the neuroblastoma gene network

The blue bars are number of genes in the network, the red line is the fitted curve. R^2 is the coefficient of determination.

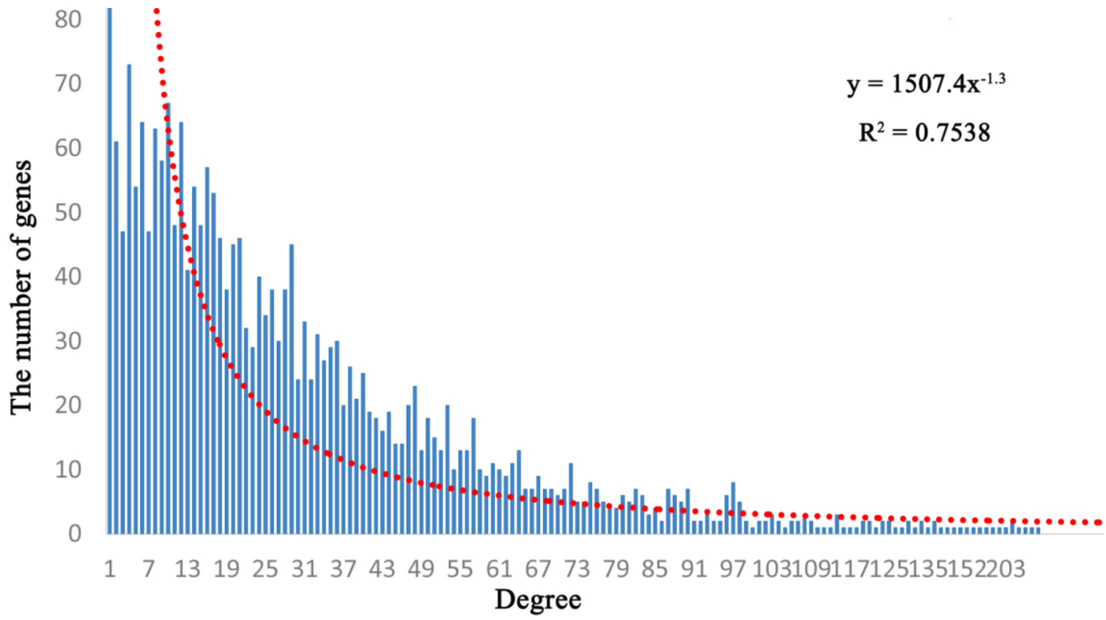

Figure S4. Gene degree distribution of the glioblastoma_seq gene network

The blue bars are number of genes in the network, the red line is the fitted curve. R^2 is the coefficient of determination.

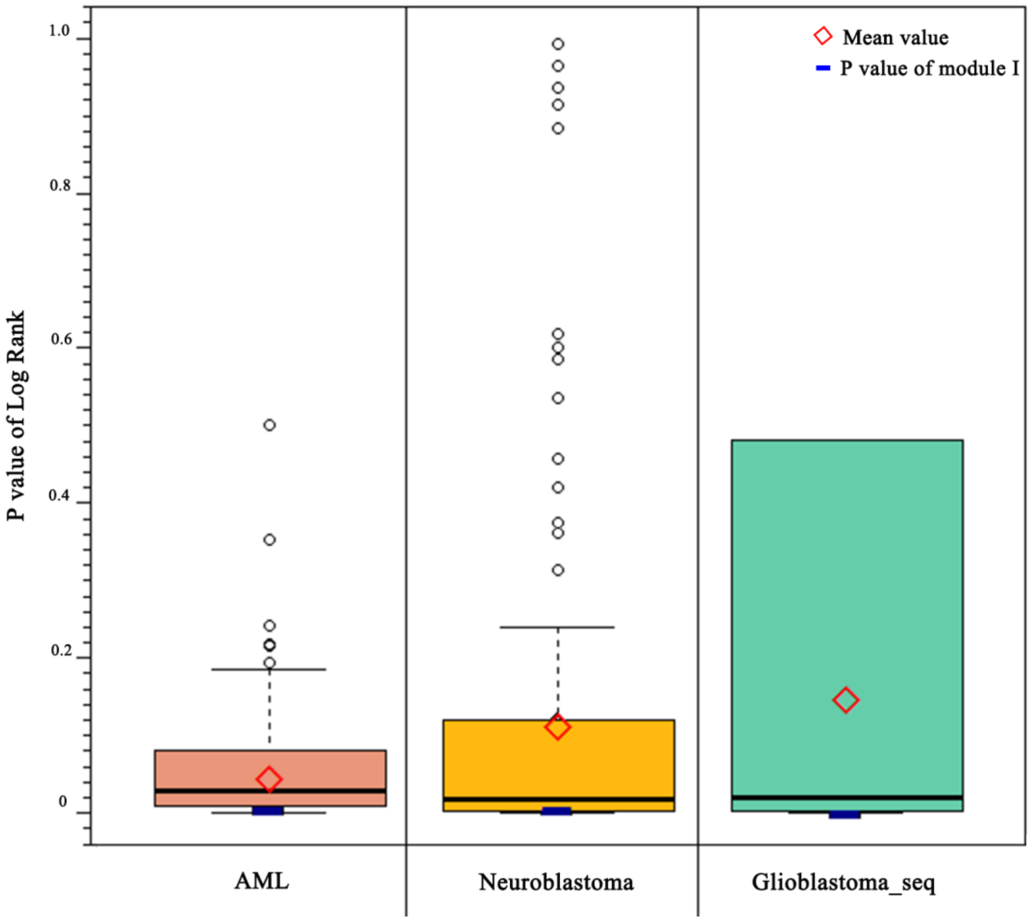

Figure S5. The log-rank p values of module I and 100 times random re-sampling for AML, neuroblastoma, and glioblastoma.
The ◇ represents mean value of the 100 times random re-sampling of module I. The _ (blue bar) represents the log-rank p value of module I for each dataset.

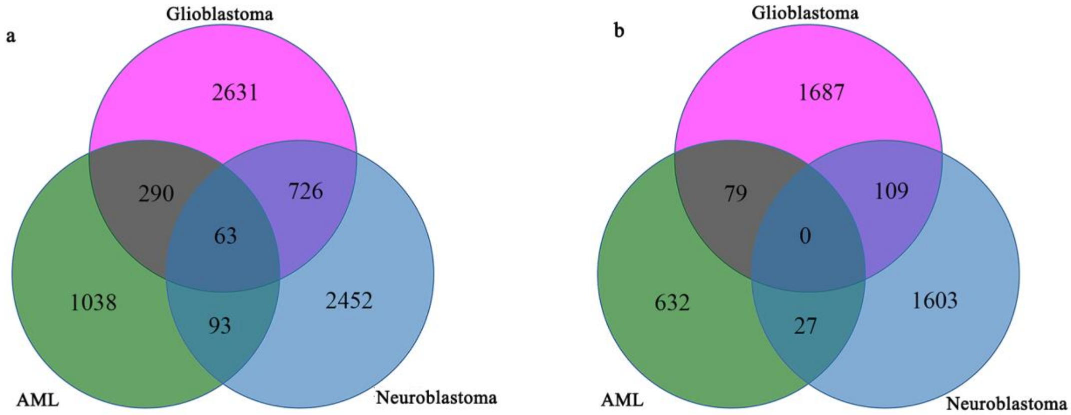

Figure S6. Genes overlap of the modules of three cancers
a. The genes overlap in three modules related to cancer initiation and progression. **b**. The genes overlap in three modules related to cellular process.

Table S1. The top 20 genes ranked by degrees in the AML network that were

used for generating the seed modules

Genesymbol	Description	Degree
ALAS2**	Catalyzing the first step of porphyrin biosynthesis	525
LAPTM4B**	Lysosomal-associated protein transmembrane 4B, among its related pathways are Lysosome	251
HDHD2*	Haloacid Dehalogenase-Like Hydrolase Domain Containing 2	237
MGC16169	It encodes a protein that contains a protein kinase domain	233
S100A12*	Regulation of inflammatory processes and immune response	222
RPL18A*	The encoded protein involved in replication of internal ribosome entry site (IRES)	191
PRKACB*	Protein kinase, cAMP-dependent, catalytic, beta subunit. Its related pathways are signaling by FGFR	191
SEPP1*	Responsible for some of the extracellular antioxidant defense properties of selenium	187
CLU*	Potentially involved in spermatogenesis. It is associated with cystadenoma and ovarian cystadenoma	183
TM7SF3*	It functions including regulation of small nuclear ribonucleoprotein biosynthesis	182
CLNS1A*	Chloride Channel, Nucleotide-Sensitive, 1A, it associates with the plasma membrane	181
CTSC*	Thiol protease. Active against of dipeptide substrates composed of both polar and hydrophobic amino acids	176
CDK6**	It’s involved in the control of the cell cycle and differentiation. Among its related pathways are PI3K-Akt signaling pathway and glioma	175
CPA3*	Highly induced by histone deacetylase inhibitor during differentiation of prostate epithelial cancer cells	172
SPINK2*	The encoded protein acts as a trypsin and acrosin inhibitor in the genital tract and is localized in the spermatozoa	171
TSC22D1*	Induced by cytokines, including TGFB1	170
SOCS2**	Regulation of cytokine signal transduction	168
FKBP3*	It encoded protein is the receptor for the two immunosuppressants which inhibit T-cell proliferation by arresting two distinct cytoplasmic signal transmission pathways	163
MGST1*	Diseases associated with MGST1 include megalencephalic leukoencephalopathy with subcortical cysts	163
C1QBP*	It is involved in inflammation and infection processes, ribosome biogenesis, regulation of apoptosis	158

** The gene was directly associated with AML.

* The gene was associated with other cancers.

Table S2. The top 20 genes ranked by degrees in the glioblastoma network that were used for generating the seed modules

Genesymbol	Description	Degree

LTF**	Lactotransferrin,80kDa,iron binding protein present in milk and a variety of secretions derived from glandular epithelium cells, it also expressed in secondary granules of neutrophils, involved in iron transport,storage and chelation, and host defense mechanisms	1698
MEOX2**	Mesodermal transcription factor that plays a key role in somitogenesis and is required for sclerotome development	787
CA3*	Diseases associated with it include hordeolum and laryngeal disease. Among its related pathways are Metabolism and Nitrogen metabolism	538
CXCL14**	Among its related pathways are TGF-Beta Pathway and TGF-Beta Pathway	475
KCNN3*	Diseases associated with it include spinocerebellar ataxia 2 and bipolar disorder	458
SOX8**	It plays a role in central nervous system, limb and facial development	434
CPLX1**	It related pathways are Transmission across Chemical Synapses and Transmission	420
NTSR2**	It is associated with G proteins that activate a phosphatidylinositol-calcium second messenger system	419
TTYH1*	Its related pathways are Ion channel transport and Transport of glucose and other sugars, bile salts and organic acids, metal ions and amine compounds	411
SERPINH1	Binds specifically to collagen. It could be involved as a chaperone in the biosynthetic pathway of collagen	399
CHI3L1**	It regulates antibacterial responses in lung by contributing to macrophage bacterial killing, controlling bacterial dissemination and augmenting host tolerance	333
CDR1*	Cerebellar degeneration-related minor antigen, 34kDa, putative neuronal signal transduction protein	314
P2RY12*	Receptor for ADP and ATP coupled to G-proteins that inhibit the adenylyl cyclase second messenger system, it is required for normal platelet aggregation and blood coagulation	311
ZNF488**	It function is sequence-specific DNA binding RNA polymerase II transcription factor activity	292
FSTL5**	Follistatin-Like 5, it is Involved in calcium ion binding	291
IL13RA2**	Diseases associated with it include glioblastoma multiform and malignant glioma	291
KNTC2*	Diseases associated with NDC80 include retinoblastoma. Among its related pathways are Cell Cycle, Mitoticand Cell Cycle, Mitotic	247
FLJ25477	Negative regulator of the canonical Wnt signaling pathway involved in neuroectodermal patterning	242
GPR17*	Dual specificity receptor for uracil nucleotides and cysteinyl leukotrienes, it may mediate brain damage by nucleotides and CysLTs following ischemia	236
ABCC3*	It acts as an inducible transporter in the biliary and intestinal excretion of organic anions, it also acts as an alternative route for the export of bile acids and glucuronides from cholestatic hepatocytes	232

** The gene was directly associated with glioblastoma.

* The gene was associated with other cancers.

Table S3. The top 20 genes ranked by degrees in the neuroblastoma network that were used for generating the seed modules

Genesymbol	Description	Degree
NTRK1**	It is involved in the development and the maturation of the central and peripheral nervous systems	959
C4orf7*	It is thought to contribute to tumor metastases by promoting cancer cell migration and invasion	658
AMIGO2*	Required for depolarization-dependent survival of cultured cerebellar granule neurons	618
CTAG1A*	It is a therapeutic target for prostate cancer	543
RP13-102H20.1	Diseases associated with it include retinitis pigmentosa 13 and prpf8-related retinitis pigmentosa. Among its related pathways are Gene Expression and Spliceosome	476
SERPINA4*	Its encoded protein product that links to apoptosis	466
TAS2R41	Receptor that may play a role in the perception of bitterness and is gustducin-linked	463
RP4-662A9.2	Diseases associated with RHO include night blindness, congenital stationary, autosomal dominant 1 and retinitis pigmentosa 4, autosomal dominant or recessive	437
CDH19**	They interact with themselves in connecting cells and contribute to the sorting of heterogeneous cell types	425
POF1B**	Plays a key role in the organization of epithelial monolayers by regulating the actin cytoskeleton	385
GPR128*	Orphan receptor	381
KRT27*	Essential for the proper assembly of type I and type II keratin protein complexes and formation of keratin intermediate filaments in the inner root sheath	364
OR2C3*	Olfactory receptors interact with odorant molecules in the nose, to initiate a neuronal response that triggers the perception of a smell	354
FLT3**	It expresses on endothelial cell, duplicated in childhood acute myeloid leukemia with poor prognosis and acute lymphoblastic leukemia but not in myelodysplastic syndrome	349
LOC402505		347
KRTAP3-3*	The protein coded by this gene is a member of the keratin-associated protein (KAP) family	343
IL1F5*	Proposed to play a role in skin inflammation	321
ITGAD*	Diseases associated with ITGAD include chronic monocytic leukemia	316
CCL19**	It plays an important role in trafficking of T cells in thymus, and in T cell and B cell migration to secondary lymphoid organs	310
LOC388002		307

** The gene was directly associated with neuroblastoma.

* The gene was associated with other cancers.

Table S4. The top 20 genes ranked by degrees in the glioblastoma_seq network that were used for generating the seed modules

Genesymbol	Description	Degree
SPOCD1	This gene encodes a protein that belongs to the TFIIS family of transcription factors. Alternate splicing results in multiple transcript variants.	252
MYBPH	Diseases associated with it include measles. Binds to myosin; probably involved in interaction with thick myofilaments in the A-band.	251
APCDD1L	A Protein Coding gene.	235
COL8A1*	Diseases associated with it include fuchs' endothelial dystrophy and macular degeneration, age-related.	227
SCNN1B*	It plays an essential role in electrolyte and blood pressure homeostasis, but also in airway surface liquid homeostasis, which is important for proper clearance of mucus.	210
PCDHGA10*	Potential calcium-dependent cell-adhesion protein. May be involved in the establishment and maintenance of specific neuronal connections in the brain.	210
MARCO**	The protein encoded by this gene is a member of the class A scavenger receptor family and is part of the innate antimicrobial immune system.	203
CHI3L1**	GO annotations related to this gene include carbohydrate binding and hydrolase activity, hydrolyzing O-glycosyl compounds.	186
NNAT**	The encoded protein of it may also play a role in forming and maintaining the structure of the nervous system. Diseases associated with NNAT include anterior horn cell disease.	176
CA9**	It may be involved in the control of cell proliferation and transformation and appears to be a novel specific biomarker for a cervical neoplasia.	174
CXCL6*	It has strong antibacterial activity against Gram-positive and Gram-negative bacteria. Diseases associated with CXCL6 include mastitis and rheumatoid arthritis.	167
ADAMTS2	It may play a role in development that is independent of its role in collagen biosynthesis. Diseases associated with it include ehlers-danlos syndrome, type viic and anosognosia.	154
COL27A1	It plays a role during the calcification of cartilage and the transition of cartilage to bone. Diseases associated with it include steel syndrome and tic disorder.	152
PTPRN*	It may be involved in processes specific for neurosecretory granules, such as their biogenesis, trafficking or regulated exocytosis or may have a general role in neuroendocrine functions.	147
SAA2**	Major acute phase reactant. Apolipoprotein of the HDL complex. Diseases associated with SAA2 include amyloidosis aa and amyloidosis, familial visceral.	143
FER1L4*	FER1L4 (Fer-1-Like Family Member 4, Pseudogene (Functional)) is a Pseudogene.	142
PI3**	This gene encodes an elastase-specific inhibitor that functions as an antimicrobial peptide against Gram-positive and Gram-negative bacteria, and fungal pathogens.	141
L1CAM**	Cell adhesion molecule with an important role in the development of the nervous system. It is involved in neuron-neuron adhesion, neurite fasciculation, outgrowth of neurites, etc.	136
COL22A1	It acts as a cell adhesion ligand for skin epithelial cells and fibroblasts.	136
IL21R*	This is a receptor for interleukin-21 , This receptor transduces the growth promoting signal	135

	of IL21, and is important for the proliferation and differentiation of T cells, B cells, and natural killer (NK) cells.

** The gene was directly associated with glioblastoma.

* The gene was associated with other cancers.

Table S5. Go and Pathway analysis of the tumor cells growth module of AML

**Category**	**Term**	***p* value**
BP(level 4)	GO:0016070~RNA metabolic process	1.36E-15
	GO:0006412~translation	3.68E-14
	GO:0044267~cellular protein metabolic process	2.29E-10
	GO:0006396~RNA processing	3.89E-10
	GO:0019538~protein metabolic process	1.70E-09
	GO:0006119~oxidative phosphorylation	6.26E-08
	GO:0051436~negative regulation of ubiquitin-protein ligase activity during mitotic cell cycle	7.13E-08
	GO:0051437~positive regulation of ubiquitin-protein ligase activity during mitotic cell cycle	1.58E-07
	GO:0051439~regulation of ubiquitin-protein ligase activity during mitotic cell cycle	3.33E-07
	GO:0051340~regulation of ligase activity	2.94E-06
	GO:0022900~electron transport chain	4.46E-06
	GO:0044271~nitrogen compound biosynthetic process	5.65E-06
	GO:0022904~respiratory electron transport chain	7.37E-06
	GO:0032269~negative regulation of cellular protein metabolic process	1.04E-05
	GO:0010605~negative regulation of macromolecule metabolic process	1.48E-05
	GO:0051248~negative regulation of protein metabolic process	2.23E-05
	GO:0006520~cellular amino acid metabolic process	2.62E-05
	GO:0043436~oxoacid metabolic process	2.62E-05
	GO:0051246~regulation of protein metabolic process	2.73E-05
	GO:0032268~regulation of cellular protein metabolic process	3.38E-05
	GO:0009892~negative regulation of metabolic process	4.60E-05
	GO:0018130~heterocycle biosynthetic process	5.11E-05
	GO:0031324~negative regulation of cellular metabolic process	1.18E-04
	GO:0044106~cellular amine metabolic process	1.28E-04
	GO:0034613~cellular protein localization	1.33E-04
	GO:0055086~nucleobase, nucleoside and nucleotide metabolic process	1.34E-04

GO:0051188~cofactor biosynthetic process	1.49E-04
GO:0046907~intracellular transport	1.50E-04
GO:0006886~intracellular protein transport	1.92E-04
GO:0015980~energy derivation by oxidation of organic compounds	2.09E-04
GO:0051247~positive regulation of protein metabolic process	2.56E-04
GO:0032270~positive regulation of cellular protein metabolic process	2.57E-04
GO:0032774~RNA biosynthetic process	2.87E-04
GO:0034622~cellular macromolecular complex assembly	3.10E-04
GO:0009165~nucleotide biosynthetic process	7.18E-04
GO:0022618~ribonucleoprotein complex assembly	0.001035
GO:0006461~protein complex assembly	0.001048
GO:0015669~gas transport	0.001137
GO:0034654~nucleobase, nucleoside, nucleotide and nucleic acid biosynthetic process	0.001249
GO:0043086~negative regulation of catalytic activity	0.001302
GO:0006779~porphyrin biosynthetic process	0.0021
GO:0034645~cellular macromolecule biosynthetic process	0.002818
GO:0006839~mitochondrial transport	0.003224
GO:0006325~chromatin organization	0.004585
GO:0010639~negative regulation of organelle organization	0.005077
GO:0015031~protein transport	0.005418
GO:0045184~establishment of protein localization	0.006748
GO:0006163~purine nucleotide metabolic process	0.006774
GO:0006259~DNA metabolic process	0.007391
GO:0044265~cellular macromolecule catabolic process	0.007785
GO:0031057~negative regulation of histone modification	0.010163
GO:0048523~negative regulation of cellular process	0.010167
GO:0015671~oxygen transport	0.010898
GO:0006732~coenzyme metabolic process	0.012514
GO:0033013~tetrapyrrole metabolic process	0.014862
GO:0006778~porphyrin metabolic process	0.014862
GO:0033044~regulation of chromosome organization	0.017487
GO:0032259~methylation	0.018345
GO:0051053~negative regulation of DNA metabolic process	0.018873
GO:0030163~protein catabolic process	0.020453
GO:0034220~ion transmembrane transport	0.021445
GO:0015985~energy coupled proton transport, down electrochemical gradient	0.021514
GO:0006767~water-soluble vitamin metabolic process	0.021514
GO:0017038~protein import	0.023904

	GO:0051129~negative regulation of cellular component organization	0.024238
	GO:0051172~negative regulation of nitrogen compound metabolic process	0.025617
	GO:0016054~organic acid catabolic process	0.026014
	GO:0015992~proton transport	0.027932
	GO:0006986~response to unfolded protein	0.028336
	GO:0006783~heme biosynthetic process	0.0289
	GO:0006349~genetic imprinting	0.029659
	GO:0006364~rRNA processing	0.029862
	GO:0045934~negative regulation of nucleobase, nucleoside, nucleotide and nucleic acid metabolic process	0.030041
	GO:0006626~protein targeting to mitochondrion	0.031134
	GO:0051169~nuclear transport	0.03239
	GO:0006818~hydrogen transport	0.033628
	GO:0016126~sterol biosynthetic process	0.035391
	GO:0006346~methylation-dependent chromatin silencing	0.04391
	GO:0006096~glycolysis	0.047341
	GO:0002377~immunoglobulin production	0.058677
	GO:0044272~sulfur compound biosynthetic process	0.062702
	GO:0001302~replicative cell aging	0.0628
	GO:0002440~production of molecular mediator of immune response	0.066035
	GO:0030100~regulation of endocytosis	0.066753
	GO:0010629~negative regulation of gene expression	0.067021
	GO:0009225~nucleotide-sugar metabolic process	0.072803
	GO:0000245~spliceosome assembly	0.073883
	GO:0010558~negative regulation of macromolecule biosynthetic process	0.076663
	GO:0042558~pteridine and derivative metabolic process	0.076857
	GO:0009890~negative regulation of biosynthetic process	0.077843
	GO:0043558~regulation of translational initiation in response to stress	0.083855
MF(level 4)	GO:0016876~ligase activity, forming aminoacyl-tRNA and related compounds	2.15E-06
	GO:0003712~transcription cofactor activity	5.14E-06
	GO:0016818~hydrolase activity, acting on acid anhydrides, in phosphorus-containing anhydrides	2.00E-05
	GO:0032555~purine ribonucleotide binding	5.83E-05
	GO:0015399~primary active transmembrane transporter activity	9.23E-05
	GO:0016655~oxidoreductase activity, acting on NADH or NADPH, quinone or similar compound as acceptor	9.81E-05
	GO:0003954~NADH dehydrogenase activity	1.25E-04

	GO:0000049~tRNA binding	3.74E-04
	GO:0016676~oxidoreductase activity, acting on heme group of donors, oxygen as acceptor	4.67E-04
	GO:0004129~cytochrome-c oxidase activity	4.67E-04
	GO:0051059~NF-kappaB binding	6.06E-04
	GO:0030554~adenyl nucleotide binding	6.92E-04
	GO:0022884~macromolecule transmembrane transporter activity	0.001286
	GO:0015450~P-P-bond-hydrolysis-driven protein transmembrane transporter activity	0.001286
	GO:0043566~structure-specific DNA binding	0.002444
	GO:0051427~hormone receptor binding	0.002841
	GO:0008320~protein transmembrane transporter activity	0.006399
	GO:0042626~ATPase activity, coupled to transmembrane movement of substances	0.008238
	GO:0016820~hydrolase activity, acting on acid anhydrides, catalyzing transmembrane movement of substances	0.009702
	GO:0003755~peptidyl-prolyl cis-trans isomerase activity	0.012522
	GO:0051287~NAD or NADH binding	0.014689
	GO:0003684~damaged DNA binding	0.020902
	GO:0051539~4 iron, 4 sulfur cluster binding	0.026534
	GO:0042974~retinoic acid receptor binding	0.026585
	GO:0008168~methyltransferase activity	0.026901
	GO:0016706~oxidoreductase activity, acting on paired donors, with incorporation or reduction of molecular oxygen, 2-oxoglutarate as one donor, and incorporation of one atom each of oxygen into both donors	0.041385
	GO:0016778~diphosphotransferase activity	0.041876
	GO:0019001~guanyl nucleotide binding	0.056198
	GO:0016881~acid-amino acid ligase activity	0.066184
	GO:0046966~thyroid hormone receptor binding	0.067676
	GO:0042809~vitamin D receptor binding	0.071297
	GO:0016747~transferase activity, transferring acyl groups other than amino-acyl groups	0.071698
	GO:0016836~hydro-lyase activity	0.082024
	GO:0042287~MHC protein binding	0.091448
KEGG Pathway	hsa05012:Parkinson's disease	5.37E-11
	hsa00190:Oxidative phosphorylation	1.59E-09
	hsa05010:Alzheimer's disease	2.00E-08
	hsa03050:Proteasome	2.03E-08
	hsa05016:Huntington's disease	1.01E-07
	hsa03040:Spliceosome	1.78E-06
	hsa03010:Ribosome	9.22E-06

	hsa00970:Aminoacyl-tRNA biosynthesis	4.89E-04
	hsa00620:Pyruvate metabolism	0.001591
	hsa00520:Amino sugar and nucleotide sugar metabolism	0.011026
	hsa00100:Steroid biosynthesis	0.011884
	hsa04260:Cardiac muscle contraction	0.014561
	hsa05340:Primary immunodeficiency	0.026815
	hsa00270:Cysteine and methionine metabolism	0.066631
	hsa00052:Galactose metabolism	0.066859
	hsa03022:Basal transcription factors	0.074869
	hsa03450:Non-homologous end-joining	0.094379

Table S6. Go and Pathway analysis of the substance biosynthesis and metabolism module of AML

Category	Term	*p* value
BP(level 4)	GO:0006412~translation	7.15E-06
	GO:0031057~negative regulation of histone modification	0.001401
	GO:0043436~oxoacid metabolic process	0.001438
	GO:0006886~intracellular protein transport	0.001875
	GO:0010639~negative regulation of organelle organization	0.002453
	GO:0034613~cellular protein localization	0.003252
	GO:0046907~intracellular transport	0.003759
	GO:0042273~ribosomal large subunit biogenesis	0.004435
	GO:0032269~negative regulation of cellular protein metabolic process	0.004978
	GO:0051248~negative regulation of protein metabolic process	0.006928
	GO:0006334~nucleosome assembly	0.009834
	GO:0016070~RNA metabolic process	0.010327
	GO:0048523~negative regulation of cellular process	0.011145
	GO:0006346~methylation-dependent chromatin silencing	0.011726
	GO:0044271~nitrogen compound biosynthetic process	0.012249
	GO:0006986~response to unfolded protein	0.012812
	GO:0015031~protein transport	0.013765
	GO:0065004~protein-DNA complex assembly	0.015489
	GO:0045184~establishment of protein localization	0.016031
	GO:0034728~nucleosome organization	0.017467
	GO:0032774~RNA biosynthetic process	0.018922
	GO:0034622~cellular macromolecular complex assembly	0.019059
	GO:0043086~negative regulation of catalytic activity	0.020406
	GO:0033044~regulation of chromosome organization	0.020734
	GO:0051130~positive regulation of cellular component organization	0.028135
	GO:0006325~chromatin organization	0.028916
	GO:0051129~negative regulation of cellular component	0.030055

organization	
GO:0010605~negative regulation of macromolecule metabolic process	0.030302
GO:0019538~protein metabolic process	0.030499
GO:0006520~cellular amino acid metabolic process	0.031168
GO:0032268~regulation of cellular protein metabolic process	0.031799
GO:0007599~hemostasis	0.038372
GO:0034645~cellular macromolecule biosynthetic process	0.039034
GO:0006898~receptor-mediated endocytosis	0.039328
GO:0000079~regulation of cyclin-dependent protein kinase activity	0.042128
GO:0007015~actin filament organization	0.042425
GO:0017038~protein import	0.043847
GO:0009892~negative regulation of metabolic process	0.044274
GO:0044106~cellular amine metabolic process	0.045775
GO:0016127~sterol catabolic process	0.046917
GO:0006349~genetic imprinting	0.046917
GO:0001666~response to hypoxia	0.049399
GO:0006631~fatty acid metabolic process	0.050271
GO:0031324~negative regulation of cellular metabolic process	0.054472
GO:0001570~vasculogenesis	0.056649
GO:0044267~cellular protein metabolic process	0.057142
GO:0001944~vasculature development	0.064523
GO:0016049~cell growth	0.065031
GO:0045931~positive regulation of mitotic cell cycle	0.065685
GO:0048522~positive regulation of cellular process	0.069527
GO:0007596~blood coagulation	0.070852
GO:0048514~blood vessel morphogenesis	0.073434
GO:0042060~wound healing	0.078733
GO:0051246~regulation of protein metabolic process	0.07986
GO:0051436~negative regulation of ubiquitin-protein ligase activity during mitotic cell cycle	0.080703
GO:0043066~negative regulation of apoptosis	0.081103
GO:0051726~regulation of cell cycle	0.082632
GO:0034654~nucleobase, nucleoside, nucleotide and nucleic acid biosynthetic process	0.083309
GO:0033043~regulation of organelle organization	0.086299
GO:0016192~vesicle-mediated transport	0.086607
GO:0006461~protein complex assembly	0.08976
GO:0043069~negative regulation of programmed cell death	0.089767
GO:0060548~negative regulation of cell death	0.091507
GO:0051437~positive regulation of ubiquitin-protein ligase	0.09366

	activity during mitotic cell cycle	
	GO:0032886~regulation of microtubule-based process	0.095453
	GO:0006839~mitochondrial transport	0.098202
	GO:0055086~nucleobase, nucleoside and nucleotide metabolic process	0.099406
	GO:0043067~regulation of programmed cell death	0.099515
MF(level 4)	GO:0051059~NF-kappaB binding	0.003301
	GO:0016818~hydrolase activity, acting on acid anhydrides, in phosphorus-containing anhydrides	0.005236
	GO:0015450~P-P-bond-hydrolysis-driven protein transmembrane transporter activity	0.009873
	GO:0022884~macromolecule transmembrane transporter activity	0.009873
	GO:0008320~protein transmembrane transporter activity	0.024693
	GO:0016876~ligase activity, forming aminoacyl-tRNA and related compounds	0.025315
	GO:0051287~NAD or NADH binding	0.025315
	GO:0032555~purine ribonucleotide binding	0.029944
	GO:0015399~primary active transmembrane transporter activity	0.030372
	GO:0070704~sterol desaturase activity	0.07008
	GO:0005070~SH3/SH2 adaptor activity	0.096534
KEGG Pathway	hsa03010:Ribosome	8.77E-07
	hsa00100:Steroid biosynthesis	0.003496
	hsa00620:Pyruvate metabolism	0.018128
	hsa00520:Amino sugar and nucleotide sugar metabolism	0.026436
	hsa04610:Complement and coagulation cascades	0.04978

Table S7. Go and Pathway analysis of the tumor proliferation and progression module of glioblastoma

Category	Term	*p* value
BP(level 4)	GO:0007399~nervous system development	8.68E-20
	GO:0048523~negative regulation of cellular process	2.96E-12
	GO:0022008~neurogenesis	1.35E-10
	GO:0010646~regulation of cell communication	3.02E-10
	GO:0019226~transmission of nerve impulse	1.87E-08
	GO:0007268~synaptic transmission	2.34E-08
	GO:0048513~organ development	3.13E-08
	GO:0007242~intracellular signaling cascade	7.89E-08
	GO:0009966~regulation of signal transduction	3.25E-07
	GO:0007417~central nervous system development	6.94E-07
	GO:0051093~negative regulation of developmental process	2.47E-06

GO:0016477~cell migration	2.59E-06
GO:0009968~negative regulation of signal transduction	4.31E-06
GO:0006811~ion transport	4.70E-06
GO:0051969~regulation of transmission of nerve impulse	6.55E-06
GO:0033273~response to vitamin	6.99E-06
GO:0048858~cell projection morphogenesis	7.49E-06
GO:0045596~negative regulation of cell differentiation	7.53E-06
GO:0048646~anatomical structure formation involved in morphogenesis	7.75E-06
GO:0048812~neuron projection morphogenesis	9.13E-06
GO:0031644~regulation of neurological system process	1.02E-05
GO:0030182~neuron differentiation	1.02E-05
GO:0032990~cell part morphogenesis	1.13E-05
GO:0010648~negative regulation of cell communication	1.20E-05
GO:0043067~regulation of programmed cell death	1.22E-05
GO:0000904~cell morphogenesis involved in differentiation	1.24E-05
GO:0045165~cell fate commitment	1.46E-05
GO:0010941~regulation of cell death	1.54E-05
GO:0048522~positive regulation of cellular process	1.68E-05
GO:0001944~vasculature development	1.90E-05
GO:0048870~cell motility	1.99E-05
GO:0022603~regulation of anatomical structure morphogenesis	2.47E-05
GO:0044057~regulation of system process	2.59E-05
GO:0001666~response to hypoxia	2.68E-05
GO:0050767~regulation of neurogenesis	2.71E-05
GO:0042060~wound healing	2.95E-05
GO:0060284~regulation of cell development	3.82E-05
GO:0000902~cell morphogenesis	3.83E-05
GO:0048666~neuron development	4.15E-05
GO:0045595~regulation of cell differentiation	4.29E-05
GO:0030155~regulation of cell adhesion	4.96E-05
GO:0001501~skeletal system development	5.32E-05
GO:0051174~regulation of phosphorus metabolic process	5.34E-05
GO:0048545~response to steroid hormone stimulus	6.94E-05
GO:0032989~cellular component morphogenesis	6.99E-05
GO:0009408~response to heat	8.35E-05
GO:0048514~blood vessel morphogenesis	9.71E-05
GO:0042127~regulation of cell proliferation	1.02E-04
GO:0006812~cation transport	3.70E-04
GO:0009952~anterior/posterior pattern formation	3.86E-04
GO:0048468~cell development	4.07E-04
GO:0051960~regulation of nervous system development	4.70E-04

GO:0009792~embryonic development ending in birth or egg hatching	4.83E-04
GO:0045778~positive regulation of ossification	5.07E-04
GO:0031667~response to nutrient levels	5.17E-04
GO:0051338~regulation of transferase activity	5.63E-04
GO:0043009~chordate embryonic development	5.66E-04
GO:0043066~negative regulation of apoptosis	5.79E-04
GO:0043069~negative regulation of programmed cell death	6.04E-04
GO:0009887~organ morphogenesis	6.07E-04
GO:0010243~response to organic nitrogen	6.46E-04
GO:0060548~negative regulation of cell death	6.66E-04
GO:0008285~negative regulation of cell proliferation	7.33E-04
GO:0031325~positive regulation of cellular metabolic process	7.64E-04
GO:0006954~inflammatory response	7.74E-04
GO:0003002~regionalization	9.23E-04
GO:0044262~cellular carbohydrate metabolic process	9.65E-04
GO:0007507~heart development	9.78E-04
GO:0007517~muscle organ development	0.001028
GO:0009893~positive regulation of metabolic process	0.001148
GO:0045667~regulation of osteoblast differentiation	0.001153
GO:0010769~regulation of cell morphogenesis involved in differentiation	0.001155
GO:0009890~negative regulation of biosynthetic process	0.001185
GO:0007420~brain development	0.001276
GO:0007156~homophilic cell adhesion	0.001358
GO:0022604~regulation of cell morphogenesis	0.001358
GO:0045665~negative regulation of neuron differentiation	0.001388
GO:0031327~negative regulation of cellular biosynthetic process	0.001406
GO:0010604~positive regulation of macromolecule metabolic process	0.001459
GO:0048488~synaptic vesicle endocytosis	0.0015
GO:0031328~positive regulation of cellular biosynthetic process	0.001827
GO:0009891~positive regulation of biosynthetic process	0.001882
GO:0048598~embryonic morphogenesis	0.001951
GO:0009892~negative regulation of metabolic process	0.002129
GO:0051173~positive regulation of nitrogen compound metabolic process	0.002138
GO:0010557~positive regulation of macromolecule biosynthetic process	0.002186
GO:0001525~angiogenesis	0.002514
GO:0031344~regulation of cell projection organization	0.002548

GO:0030501~positive regulation of bone mineralization	0.002592
GO:0043412~biopolymer modification	0.002643
GO:0035239~tube morphogenesis	0.002695
GO:0010558~negative regulation of macromolecule biosynthetic process	0.00281
GO:0030278~regulation of ossification	0.003199
GO:0030036~actin cytoskeleton organization	0.003437
GO:0031324~negative regulation of cellular metabolic process	0.003477
GO:0005996~monosaccharide metabolic process	0.003633
GO:0043244~regulation of protein complex disassembly	0.003701
GO:0007599~hemostasis	0.003792
GO:0030199~collagen fibril organization	0.003868
GO:0051253~negative regulation of RNA metabolic process	0.004113
GO:0070169~positive regulation of biomineral formation	0.004116
GO:0050982~detection of mechanical stimulus	0.004507
GO:0050974~detection of mechanical stimulus involved in sensory perception	0.004692
GO:0006937~regulation of muscle contraction	0.004795
GO:0045785~positive regulation of cell adhesion	0.004842
GO:0001764~neuron migration	0.004856
GO:0007160~cell-matrix adhesion	0.005257
GO:0030500~regulation of bone mineralization	0.005367
GO:0007596~blood coagulation	0.005571
GO:0010605~negative regulation of macromolecule metabolic process	0.005717
GO:0051254~positive regulation of RNA metabolic process	0.005846
GO:0007411~axon guidance	0.006224
GO:0010038~response to metal ion	0.006548
GO:0045935~positive regulation of nucleobase, nucleoside, nucleotide and nucleic acid metabolic process	0.006776
GO:0007165~signal transduction	0.006978
GO:0019318~hexose metabolic process	0.007023
GO:0010975~regulation of neuron projection development	0.00709
GO:0014070~response to organic cyclic substance	0.007214
GO:0050905~neuromuscular process	0.00729
GO:0000079~regulation of cyclin-dependent protein kinase activity	0.007317
GO:0010629~negative regulation of gene expression	0.007445
GO:0048266~behavioral response to pain	0.007672
GO:0051270~regulation of cell motion	0.0078
GO:0051240~positive regulation of multicellular organismal process	0.008192

GO:0043086~negative regulation of catalytic activity	0.008499
GO:0051493~regulation of cytoskeleton organization	0.009127
GO:0010628~positive regulation of gene expression	0.00946
GO:0070167~regulation of biomineral formation	0.009721
GO:0051172~negative regulation of nitrogen compound metabolic process	0.010317
GO:0043068~positive regulation of programmed cell death	0.011171
GO:0051259~protein oligomerization	0.011511
GO:0045934~negative regulation of nucleobase, nucleoside, nucleotide and nucleic acid metabolic process	0.012107
GO:0031099~regeneration	0.012395
GO:0045792~negative regulation of cell size	0.012659
GO:0048489~synaptic vesicle transport	0.012731
GO:0010942~positive regulation of cell death	0.012741
GO:0006022~aminoglycan metabolic process	0.012758
GO:0043242~negative regulation of protein complex disassembly	0.013581
GO:0030097~hemopoiesis	0.013894
GO:0016192~vesicle-mediated transport	0.016298
GO:0006897~endocytosis	0.017537
GO:0017156~calcium ion-dependent exocytosis	0.017859
GO:0060562~epithelial tube morphogenesis	0.017929
GO:0006796~phosphate metabolic process	0.018246
GO:0032870~cellular response to hormone stimulus	0.018345
GO:0045765~regulation of angiogenesis	0.018582
GO:0031098~stress-activated protein kinase signaling pathway	0.018582
GO:0019725~cellular homeostasis	0.019212
GO:0043085~positive regulation of catalytic activity	0.019221
GO:0033043~regulation of organelle organization	0.020254
GO:0009311~oligosaccharide metabolic process	0.020319
GO:0022037~metencephalon development	0.020826
GO:0001838~embryonic epithelial tube formation	0.020927
GO:0048534~hemopoietic or lymphoid organ development	0.021121
GO:0030866~cortical actin cytoskeleton organization	0.021804
GO:0034612~response to tumor necrosis factor	0.021804
GO:0048265~response to pain	0.021832
GO:0010647~positive regulation of cell communication	0.024263
GO:0010720~positive regulation of cell development	0.0246
GO:0010811~positive regulation of cell-substrate adhesion	0.025218
GO:0035148~tube lumen formation	0.025562
GO:0048863~stem cell differentiation	0.025871
GO:0016052~carbohydrate catabolic process	0.025894
GO:0009888~tissue development	0.026214

GO:0060627~regulation of vesicle-mediated transport	0.027211
GO:0030334~regulation of cell migration	0.027275
GO:0009100~glycoprotein metabolic process	0.027866
GO:0030308~negative regulation of cell growth	0.028777
GO:0045926~negative regulation of growth	0.029077
GO:0051129~negative regulation of cellular component organization	0.029333
GO:0046928~regulation of neurotransmitter secretion	0.029628
GO:0032268~regulation of cellular protein metabolic process	0.029855
GO:0051049~regulation of transport	0.030379
GO:0001503~ossification	0.030674
GO:0001558~regulation of cell growth	0.031361
GO:0045669~positive regulation of osteoblast differentiation	0.031374
GO:0019827~stem cell maintenance	0.031374
GO:0030865~cortical cytoskeleton organization	0.032245
GO:0048662~negative regulation of smooth muscle cell proliferation	0.032245
GO:0005976~polysaccharide metabolic process	0.032552
GO:0046903~secretion	0.033349
GO:0002520~immune system development	0.03366
GO:0060537~muscle tissue development	0.033711
GO:0008344~adult locomotory behavior	0.034284
GO:0006873~cellular ion homeostasis	0.035402
GO:0051101~regulation of DNA binding	0.035811
GO:0048732~gland development	0.036535
GO:0007050~cell cycle arrest	0.036674
GO:0010810~regulation of cell-substrate adhesion	0.037038
GO:0001649~osteoblast differentiation	0.038271
GO:0051591~response to cAMP	0.038271
GO:0060324~face development	0.038597
GO:0043254~regulation of protein complex assembly	0.039004
GO:0032271~regulation of protein polymerization	0.040049
GO:0048864~stem cell development	0.04026
GO:0031100~organ regeneration	0.04026
GO:0045768~positive regulation of anti-apoptosis	0.040487
GO:0021549~cerebellum development	0.040487
GO:0060606~tube closure	0.040487
GO:0002237~response to molecule of bacterial origin	0.041372
GO:0051246~regulation of protein metabolic process	0.043094
GO:0006096~glycolysis	0.043983
GO:0007612~learning	0.044536
GO:0051893~regulation of focal adhesion formation	0.044917

GO:0008347~glial cell migration	0.044917
GO:0035313~wound healing, spreading of epidermal cells	0.044917
GO:0070085~glycosylation	0.045507
GO:0006486~protein amino acid glycosylation	0.045507
GO:0007162~negative regulation of cell adhesion	0.045787
GO:0048729~tissue morphogenesis	0.046841
GO:0014075~response to amine stimulus	0.047472
GO:0007613~memory	0.047472
GO:0016188~synaptic vesicle maturation	0.048255
GO:0046469~platelet activating factor metabolic process	0.048255
GO:0032964~collagen biosynthetic process	0.048255
GO:0045087~innate immune response	0.048526
GO:0048483~autonomic nervous system development	0.048996
GO:0021545~cranial nerve development	0.048996
GO:0042176~regulation of protein catabolic process	0.049326
GO:0006836~neurotransmitter transport	0.049546
GO:0060341~regulation of cellular localization	0.049812
GO:0007423~sensory organ development	0.050935
GO:0006576~biogenic amine metabolic process	0.05172
GO:0051651~maintenance of location in cell	0.051793
GO:0051094~positive regulation of developmental process	0.052621
GO:0008354~germ cell migration	0.056233
GO:0016051~carbohydrate biosynthetic process	0.056273
GO:0048878~chemical homeostasis	0.056276
GO:0001841~neural tube formation	0.056701
GO:0032956~regulation of actin cytoskeleton organization	0.058596
GO:0048565~gut development	0.059016
GO:0006986~response to unfolded protein	0.05946
GO:0007163~establishment or maintenance of cell polarity	0.060508
GO:0008284~positive regulation of cell proliferation	0.061547
GO:0031114~regulation of microtubule depolymerization	0.061574
GO:0031532~actin cytoskeleton reorganization	0.061574
GO:0007026~negative regulation of microtubule depolymerization	0.061574
GO:0045598~regulation of fat cell differentiation	0.061574
GO:0051604~protein maturation	0.062216
GO:0008299~isoprenoid biosynthetic process	0.063622
GO:0051495~positive regulation of cytoskeleton organization	0.063756
GO:0051726~regulation of cell cycle	0.065951
GO:0032270~positive regulation of cellular protein metabolic process	0.066549
GO:0001570~vasculogenesis	0.067057
GO:0050818~regulation of coagulation	0.067057

GO:0032507~maintenance of protein location in cell	0.067057
GO:0051271~negative regulation of cell motion	0.067196
GO:0060325~face morphogenesis	0.069153
GO:0006575~cellular amino acid derivative metabolic process	0.069585
GO:0051971~positive regulation of transmission of nerve impulse	0.070355
GO:0009895~negative regulation of catabolic process	0.070355
GO:0032496~response to lipopolysaccharide	0.070852
GO:0051130~positive regulation of cellular component organization	0.072658
GO:0014020~primary neural tube formation	0.073558
GO:0021987~cerebral cortex development	0.073558
GO:0006325~chromatin organization	0.07433
GO:0051494~negative regulation of cytoskeleton organization	0.075964
GO:0006887~exocytosis	0.07732
GO:0008045~motor axon guidance	0.07775
GO:0002009~morphogenesis of an epithelium	0.077841
GO:0048663~neuron fate commitment	0.078572
GO:0051099~positive regulation of binding	0.079231
GO:0009967~positive regulation of signal transduction	0.079973
GO:0006915~apoptosis	0.080098
GO:0032970~regulation of actin filament-based process	0.080336
GO:0006461~protein complex assembly	0.080374
GO:0035270~endocrine system development	0.080587
GO:0042107~cytokine metabolic process	0.080645
GO:0070555~response to interleukin-1	0.080645
GO:0042177~negative regulation of protein catabolic process	0.080645
GO:0042552~myelination	0.082996
GO:0060443~mammary gland morphogenesis	0.083299
GO:0045071~negative regulation of viral genome replication	0.083299
GO:0001763~morphogenesis of a branching structure	0.084583
GO:0048754~branching morphogenesis of a tube	0.086008
GO:0017157~regulation of exocytosis	0.087495
GO:0007269~neurotransmitter secretion	0.087495
GO:0034976~response to endoplasmic reticulum stress	0.087495
GO:0002521~leukocyte differentiation	0.089859
GO:0016053~organic acid biosynthetic process	0.089871
GO:0051247~positive regulation of protein metabolic process	0.091003
GO:0042330~taxis	0.091022

	GO:0006935~chemotaxis	0.091022
	GO:0009855~determination of bilateral symmetry	0.091263
	GO:0009799~determination of symmetry	0.091263
	GO:0060137~maternal process involved in parturition	0.091631
	GO:0002576~platelet degranulation	0.091631
	GO:0007566~embryo implantation	0.091966
	GO:0060173~limb development	0.093777
	GO:0008610~lipid biosynthetic process	0.094342
	GO:0051588~regulation of neurotransmitter transport	0.096231
	GO:0048278~vesicle docking	0.096231
	GO:0031646~positive regulation of neurological system process	0.096952
	GO:0045185~maintenance of protein location	0.098522
	GO:0010721~negative regulation of cell development	0.098522
	GO:0035282~segmentation	0.098522
	GO:0046685~response to arsenic	0.098721
	GO:0021680~cerebellar Purkinje cell layer development	0.098721
	GO:0007413~axonal fasciculation	0.098721
	GO:0051647~nucleus localization	0.098721
MF(level 4)	GO:0015267~channel activity	1.05E-06
	GO:0015075~ion transmembrane transporter activity	1.55E-05
	GO:0042803~protein homodimerization activity	9.98E-04
	GO:0008194~UDP-glycosyltransferase activity	0.001834
	GO:0003779~actin binding	0.002622
	GO:0005085~guanyl-nucleotide exchange factor activity	0.002841
	GO:0005539~glycosaminoglycan binding	0.009957
	GO:0046872~metal ion binding	0.010289
	GO:0042578~phosphoric ester hydrolase activity	0.010916
	GO:0016758~transferase activity, transferring hexosyl groups	0.013366
	GO:0005159~insulin-like growth factor receptor binding	0.014521
	GO:0019905~syntaxin binding	0.021621
	GO:0043394~proteoglycan binding	0.022733
	GO:0046923~ER retention sequence binding	0.022976
	GO:0005095~GTPase inhibitor activity	0.025718
	GO:0019894~kinesin binding	0.025718
	GO:0003712~transcription cofactor activity	0.029549
	GO:0016301~kinase activity	0.038976
	GO:0016763~transferase activity, transferring pentosyl groups	0.039598
	GO:0048407~platelet-derived growth factor binding	0.039966
	GO:0005178~integrin binding	0.041337
	GO:0033558~protein deacetylase activity	0.047245
	GO:0046982~protein heterodimerization activity	0.051021

	GO:0002020~protease binding	0.051108
	GO:0016864~intramolecular oxidoreductase activity, transposing S-S bonds	0.071117
	GO:0016298~lipase activity	0.076766
	GO:0017124~SH3 domain binding	0.081631
	GO:0008601~protein phosphatase type 2A regulator activity	0.08357
	GO:0008483~transaminase activity	0.083842
	GO:0017091~AU-rich element binding	0.08519
	GO:0005121~Toll binding	0.09316
	GO:0005342~organic acid transmembrane transporter activity	0.095679
	GO:0004866~endopeptidase inhibitor activity	0.095995
KEGG Pathway	hsa04512:ECM-receptor interaction	8.79E-06
	hsa04510:Focal adhesion	1.35E-05
	hsa04310:Wnt signaling pathway	4.73E-05
	hsa04720:Long-term potentiation	8.89E-05
	hsa05412:Arrhythmogenic right ventricular cardiomyopathy (ARVC)	3.26E-04
	hsa04010:MAPK signaling pathway	4.31E-04
	hsa00512:O-Glycan biosynthesis	0.001236
	hsa04916:Melanogenesis	0.002085
	hsa04020:Calcium signaling pathway	0.002928
	hsa04360:Axon guidance	0.003398
	hsa05200:Pathways in cancer	0.003481
	hsa04730:Long-term depression	0.004411
	hsa05414:Dilated cardiomyopathy	0.004707
	hsa04912:GnRH signaling pathway	0.00677
	hsa00511:Other glycan degradation	0.008562
	hsa05210:Colorectal cancer	0.008799
	hsa05410:Hypertrophic cardiomyopathy (HCM)	0.010426
	hsa04080:Neuroactive ligand-receptor interaction	0.012237
	hsa04115:p53 signaling pathway	0.015775
	hsa04610:Complement and coagulation cascades	0.018772
	hsa00052:Galactose metabolism	0.028618
	hsa04810:Regulation of actin cytoskeleton	0.031383
	hsa04520:Adherens junction	0.035243
	hsa04210:Apoptosis	0.045155
	hsa00030:Pentose phosphate pathway	0.056081
	hsa00604:Glycosphingolipid biosynthesis	0.07006
	hsa04530:Tight junction	0.074207
	hsa00051:Fructose and mannose metabolism	0.07541
	hsa05217:Basal cell carcinoma	0.087444

	hsa04330:Notch signaling pathway	0.089632
	hsa00120:Primary bile acid biosynthesis	0.093206

Table S8. Go and Pathway analysis of the cellular signaling transduction module of glioblastoma

Category	Term	*p* value
BP(level 4)	GO:0007399~nervous system development	1.42E-10
	GO:0007242~intracellular signaling cascade	3.60E-07
	GO:0007268~synaptic transmission	5.64E-07
	GO:0022008~neurogenesis	5.27E-06
	GO:0048523~negative regulation of cellular process	8.28E-06
	GO:0019318~hexose metabolic process	1.07E-05
	GO:0010646~regulation of cell communication	1.12E-05
	GO:0006811~ion transport	1.30E-05
	GO:0019226~transmission of nerve impulse	2.11E-05
	GO:0048522~positive regulation of cellular process	2.33E-05
	GO:0048545~response to steroid hormone stimulus	2.58E-05
	GO:0005996~monosaccharide metabolic process	2.76E-05
	GO:0045595~regulation of cell differentiation	2.87E-05
	GO:0044057~regulation of system process	3.65E-05
	GO:0006812~cation transport	5.85E-05
	GO:0044262~cellular carbohydrate metabolic process	1.11E-04
	GO:0051093~negative regulation of developmental process	1.13E-04
	GO:0042127~regulation of cell proliferation	1.48E-04
	GO:0045596~negative regulation of cell differentiation	1.56E-04
	GO:0016202~regulation of striated muscle tissue development	1.71E-04
	GO:0048634~regulation of muscle development	2.16E-04
	GO:0051726~regulation of cell cycle	2.23E-04
	GO:0051329~interphase of mitotic cell cycle	2.41E-04
	GO:0045165~cell fate commitment	2.67E-04
	GO:0010001~glial cell differentiation	3.34E-04
	GO:0051325~interphase	3.70E-04
	GO:0016051~carbohydrate biosynthetic process	4.24E-04
	GO:0007417~central nervous system development	4.71E-04
	GO:0031099~regeneration	6.39E-04
	GO:0009966~regulation of signal transduction	7.65E-04
	GO:0043068~positive regulation of programmed cell death	7.67E-04
	GO:0051247~positive regulation of protein metabolic process	8.60E-04
	GO:0010942~positive regulation of cell death	8.63E-04
	GO:0060284~regulation of cell development	0.001006
	GO:0001501~skeletal system development	0.001016

	GO:0030182~neuron differentiation	0.00103
	GO:0008284~positive regulation of cell proliferation	0.001143
	GO:0051174~regulation of phosphorus metabolic process	0.001257
	GO:0031644~regulation of neurological system process	0.00126
	GO:0010648~negative regulation of cell communication	0.001274
	GO:0007165~signal transduction	0.001333
	GO:0043067~regulation of programmed cell death	0.001491
	GO:0010941~regulation of cell death	0.00168
	GO:0005976~polysaccharide metabolic process	0.00174
	GO:0048265~response to pain	0.001816
	GO:0009968~negative regulation of signal transduction	0.001948
	GO:0000082~G1/S transition of mitotic cell cycle	0.001982
	GO:0014070~response to organic cyclic substance	0.00232
	GO:0010038~response to metal ion	0.002432
	GO:0032270~positive regulation of cellular protein metabolic process	0.002609
	GO:0001558~regulation of cell growth	0.002975
	GO:0051969~regulation of transmission of nerve impulse	0.00314
	GO:0048666~neuron development	0.003597
	GO:0044264~cellular polysaccharide metabolic process	0.003632
	GO:0044246~regulation of multicellular organismal metabolic process	0.003692
	GO:0032268~regulation of cellular protein metabolic process	0.004341
	GO:0000302~response to reactive oxygen species	0.004381
	GO:0043393~regulation of protein binding	0.004696
	GO:0022403~cell cycle phase	0.004776
	GO:0051960~regulation of nervous system development	0.004807
	GO:0016477~cell migration	0.004992
	GO:0051270~regulation of cell motion	0.005187
	GO:0006259~DNA metabolic process	0.005474
	GO:0048513~organ development	0.005508
	GO:0051049~regulation of transport	0.005618
	GO:0051094~positive regulation of developmental process	0.00565
	GO:0022603~regulation of anatomical structure morphogenesis	0.005668
	GO:0048468~cell development	0.005757
	GO:0010243~response to organic nitrogen	0.005869
	GO:0007156~homophilic cell adhesion	0.006259
	GO:0009952~anterior/posterior pattern formation	0.006902
	GO:0010604~positive regulation of macromolecule metabolic process	0.007754
	GO:0000271~polysaccharide biosynthetic process	0.008757
	GO:0031100~organ regeneration	0.00927

	GO:0051246~regulation of protein metabolic process	0.009471
	GO:0006915~apoptosis	0.009857
	GO:0009893~positive regulation of metabolic process	0.009988
	GO:0045665~negative regulation of neuron differentiation	0.010787
	GO:0051272~positive regulation of cell motion	0.011047
	GO:0009792~embryonic development ending in birth or egg hatching	0.011398
	GO:0031325~positive regulation of cellular metabolic process	0.011594
	GO:0000075~cell cycle checkpoint	0.011857
	GO:0048638~regulation of developmental growth	0.011942
	GO:0034637~cellular carbohydrate biosynthetic process	0.012709
	GO:0051338~regulation of transferase activity	0.012789
	GO:0045637~regulation of myeloid cell differentiation	0.014283
	GO:0043085~positive regulation of catalytic activity	0.014436
	GO:0042133~neurotransmitter metabolic process	0.014714
	GO:0050767~regulation of neurogenesis	0.014782
	GO:0043009~chordate embryonic development	0.015222
	GO:0002237~response to molecule of bacterial origin	0.015596
	GO:0060043~regulation of cardiac muscle cell proliferation	0.015828
	GO:0055021~regulation of cardiac muscle growth	0.015828
	GO:0032886~regulation of microtubule-based process	0.015931
	GO:0070507~regulation of microtubule cytoskeleton organization	0.015943
	GO:0001666~response to hypoxia	0.015944
	GO:0051173~positive regulation of nitrogen compound metabolic process	0.01603
	GO:0045471~response to ethanol	0.0169
	GO:0048870~cell motility	0.017414
	GO:0008285~negative regulation of cell proliferation	0.017711
	GO:0007346~regulation of mitotic cell cycle	0.017871
	GO:0006937~regulation of muscle contraction	0.017876
	GO:0030334~regulation of cell migration	0.018203
	GO:0009165~nucleotide biosynthetic process	0.018301
	GO:0045776~negative regulation of blood pressure	0.018336
	GO:0045597~positive regulation of cell differentiation	0.018361
	GO:0051493~regulation of cytoskeleton organization	0.018614
	GO:0009894~regulation of catabolic process	0.019248
	GO:0006796~phosphate metabolic process	0.019286
	GO:0030155~regulation of cell adhesion	0.020074
	GO:0031344~regulation of cell projection organization	0.020904
	GO:0060420~regulation of heart growth	0.021977
	GO:0010712~regulation of collagen metabolic process	0.021977

	GO:0010564~regulation of cell cycle process	0.022756
	GO:0051130~positive regulation of cellular component organization	0.022906
	GO:0040017~positive regulation of locomotion	0.023034
	GO:0019915~lipid storage	0.023549
	GO:0043412~biopolymer modification	0.023726
	GO:0044255~cellular lipid metabolic process	0.025849
	GO:0009887~organ morphogenesis	0.025965
	GO:0007420~brain development	0.026629
	GO:0044271~nitrogen compound biosynthetic process	0.026715
	GO:0055086~nucleobase, nucleoside and nucleotide metabolic process	0.026737
	GO:0010562~positive regulation of phosphorus metabolic process	0.027355
	GO:0034654~nucleobase, nucleoside, nucleotide and nucleic acid biosynthetic process	0.02814
	GO:0031329~regulation of cellular catabolic process	0.028278
	GO:0007613~memory	0.028734
	GO:0043066~negative regulation of apoptosis	0.028961
	GO:0031109~microtubule polymerization or depolymerization	0.029394
	GO:0042219~cellular amino acid derivative catabolic process	0.029511
	GO:0032496~response to lipopolysaccharide	0.029805
	GO:0000079~regulation of cyclin-dependent protein kinase activity	0.030113
	GO:0050905~neuromuscular process	0.031518
	GO:0051051~negative regulation of transport	0.031714
	GO:0031346~positive regulation of cell projection organization	0.03176
	GO:0045639~positive regulation of myeloid cell differentiation	0.033533
	GO:0051494~negative regulation of cytoskeleton organization	0.03377
	GO:0055093~response to hyperoxia	0.033788
	GO:0003002~regionalization	0.035349
	GO:0031667~response to nutrient levels	0.035349
	GO:0043069~negative regulation of programmed cell death	0.035538
	GO:0008610~lipid biosynthetic process	0.036408
	GO:0001944~vasculature development	0.036867
	GO:0060548~negative regulation of cell death	0.036951
	GO:0051254~positive regulation of RNA metabolic process	0.037288

	GO:0043242~negative regulation of protein complex disassembly	0.037708
	GO:0002761~regulation of myeloid leukocyte differentiation	0.037708
	GO:0016053~organic acid biosynthetic process	0.038509
	GO:0010827~regulation of glucose transport	0.038868
	GO:0045935~positive regulation of nucleobase, nucleoside, nucleotide and nucleic acid metabolic process	0.03927
	GO:0051241~negative regulation of multicellular organismal process	0.039364
	GO:0033043~regulation of organelle organization	0.040076
	GO:0042060~wound healing	0.041201
	GO:0048704~embryonic skeletal system morphogenesis	0.04199
	GO:0050900~leukocyte migration	0.04199
	GO:0030335~positive regulation of cell migration	0.042369
	GO:0048663~neuron fate commitment	0.042829
	GO:0030307~positive regulation of cell growth	0.042829
	GO:0045786~negative regulation of cell cycle	0.042862
	GO:0015804~neutral amino acid transport	0.044069
	GO:0051169~nuclear transport	0.04617
	GO:0007067~mitosis	0.046618
	GO:0000280~nuclear division	0.046618
	GO:0010639~negative regulation of organelle organization	0.046669
	GO:0033273~response to vitamin	0.047116
	GO:0046325~negative regulation of glucose import	0.047152
	GO:0042135~neurotransmitter catabolic process	0.047152
	GO:0044252~negative regulation of multicellular organismal metabolic process	0.048041
	GO:0010713~negative regulation of collagen metabolic process	0.048041
	GO:0009448~gamma-aminobutyric acid metabolic process	0.048041
	GO:0046627~negative regulation of insulin receptor signaling pathway	0.04816
	GO:0043269~regulation of ion transport	0.048565
	GO:0001708~cell fate specification	0.050479
	GO:0010638~positive regulation of organelle organization	0.050704
	GO:0060541~respiratory system development	0.050775
	GO:0030193~regulation of blood coagulation	0.051128
	GO:0009892~negative regulation of metabolic process	0.055021
	GO:0055085~transmembrane transport	0.056312
	GO:0000087~M phase of mitotic cell cycle	0.05652
	GO:0032271~regulation of protein polymerization	0.056602
	GO:0045785~positive regulation of cell adhesion	0.056718
	GO:0009895~negative regulation of catabolic process	0.058076

	GO:0006631~fatty acid metabolic process	0.059305
	GO:0043470~regulation of carbohydrate catabolic process	0.059516
	GO:0051148~negative regulation of muscle cell differentiation	0.059516
	GO:0043471~regulation of cellular carbohydrate catabolic process	0.059516
	GO:0010769~regulation of cell morphogenesis involved in differentiation	0.060798
	GO:0008016~regulation of heart contraction	0.060798
	GO:0051495~positive regulation of cytoskeleton organization	0.060862
	GO:0043467~regulation of generation of precursor metabolites and energy	0.061027
	GO:0010720~positive regulation of cell development	0.061788
	GO:0050880~regulation of blood vessel size	0.062097
	GO:0043271~negative regulation of ion transport	0.062249
	GO:0010829~negative regulation of glucose transport	0.062696
	GO:0031324~negative regulation of cellular metabolic process	0.065549
	GO:0010675~regulation of cellular carbohydrate metabolic process	0.065578
	GO:0009310~amine catabolic process	0.065897
	GO:0000226~microtubule cytoskeleton organization	0.067192
	GO:0010975~regulation of neuron projection development	0.067275
	GO:0048660~regulation of smooth muscle cell proliferation	0.067787
	GO:0010557~positive regulation of macromolecule biosynthetic process	0.069594
	GO:0008637~apoptotic mitochondrial changes	0.069823
	GO:0006633~fatty acid biosynthetic process	0.071267
	GO:0048514~blood vessel morphogenesis	0.071631
	GO:0060341~regulation of cellular localization	0.072979
	GO:0050678~regulation of epithelial cell proliferation	0.073068
	GO:0022604~regulation of cell morphogenesis	0.0736
	GO:0006109~regulation of carbohydrate metabolic process	0.073638
	GO:0014075~response to amine stimulus	0.073638
	GO:0051642~centrosome localization	0.07506
	GO:0060045~positive regulation of cardiac muscle cell proliferation	0.07506
	GO:0045927~positive regulation of growth	0.076912
	GO:0010628~positive regulation of gene expression	0.077086
	GO:0048863~stem cell differentiation	0.079312
	GO:0042246~tissue regeneration	0.079312
	GO:0006163~purine nucleotide metabolic process	0.079415

	GO:0032370~positive regulation of lipid transport	0.080271
	GO:0021895~cerebral cortex neuron differentiation	0.080271
	GO:0051050~positive regulation of transport	0.081333
	GO:0009408~response to heat	0.08248
	GO:0051651~maintenance of location in cell	0.083033
	GO:0045793~positive regulation of cell size	0.083033
	GO:0051129~negative regulation of cellular component organization	0.08311
	GO:0050772~positive regulation of axonogenesis	0.084053
	GO:0021515~cell differentiation in spinal cord	0.084053
	GO:0007411~axon guidance	0.084144
	GO:0032024~positive regulation of insulin secretion	0.086049
	GO:0043436~oxoacid metabolic process	0.086432
	GO:0030324~lung development	0.087873
	GO:0051240~positive regulation of multicellular organismal process	0.090365
	GO:0000904~cell morphogenesis involved in differentiation	0.090365
	GO:0010605~negative regulation of macromolecule metabolic process	0.09141
	GO:0050679~positive regulation of epithelial cell proliferation	0.091426
	GO:0050818~regulation of coagulation	0.091426
	GO:0031328~positive regulation of cellular biosynthetic process	0.091867
	GO:0000279~M phase	0.092237
	GO:0006936~muscle contraction	0.092507
	GO:0009891~positive regulation of biosynthetic process	0.092719
	GO:0003018~vascular process in circulatory system	0.098062
	GO:0051926~negative regulation of calcium ion transport	0.099702
MF(level 4)	GO:0015267~channel activity	4.26E-06
	GO:0015075~ion transmembrane transporter activity	7.93E-06
	GO:0042803~protein homodimerization activity	2.97E-04
	GO:0016301~kinase activity	0.003525
	GO:0016773~phosphotransferase activity, alcohol group as acceptor	0.010153
	GO:0008146~sulfotransferase activity	0.013814
	GO:0005085~guanyl-nucleotide exchange factor activity	0.014073
	GO:0019905~syntaxin binding	0.021883
	GO:0005096~GTPase activator activity	0.022636
	GO:0042626~ATPase activity, coupled to transmembrane movement of substances	0.02434
	GO:0070279~vitamin B6 binding	0.026357
	GO:0030170~pyridoxal phosphate binding	0.026357

	GO:0016820~hydrolase activity, acting on acid anhydrides, catalyzing transmembrane movement of substances	0.028536
	GO:0043566~structure-specific DNA binding	0.02858
	GO:0042578~phosphoric ester hydrolase activity	0.028617
	GO:0046982~protein heterodimerization activity	0.031255
	GO:0015399~primary active transmembrane transporter activity	0.034507
	GO:0016298~lipase activity	0.044624
	GO:0033558~protein deacetylase activity	0.044981
	GO:0046923~ER retention sequence binding	0.046133
	GO:0070053~thrombospondin receptor activity	0.046133
	GO:0005083~small GTPase regulator activity	0.053326
	GO:0005539~glycosaminoglycan binding	0.062669
	GO:0003779~actin binding	0.063437
	GO:0045296~cadherin binding	0.067617
	GO:0009374~biotin binding	0.072182
	GO:0015631~tubulin binding	0.081665
	GO:0043565~sequence-specific DNA binding	0.081853
	GO:0004879~ligand-dependent nuclear receptor activity	0.088368
	GO:0016758~transferase activity, transferring hexosyl groups	0.089033
	GO:0005342~organic acid transmembrane transporter activity	0.091856
	GO:0005159~insulin-like growth factor receptor binding	0.09475
KEGG Pathway	hsa04020:Calcium signaling pathway	1.82E-04
	hsa04720:Long-term potentiation	9.63E-04
	hsa05200:Pathways in cancer	0.002882
	hsa04930:Type II diabetes mellitus	0.004364
	hsa04080:Neuroactive ligand-receptor interaction	0.005824
	hsa00250:Alanine, aspartate and glutamate metabolism	0.017526
	hsa04730:Long-term depression	0.018166
	hsa05210:Colorectal cancer	0.021458
	hsa05014:Amyotrophic lateral sclerosis (ALS)	0.029295
	hsa04010:MAPK signaling pathway	0.029502
	hsa04070:Phosphatidylinositol signaling system	0.03192
	hsa04115:p53 signaling pathway	0.035087
	hsa05110:Vibrio cholerae infection	0.042214
	hsa05222:Small cell lung cancer	0.042777
	hsa04360:Axon guidance	0.046836
	hsa04114:Oocyte meiosis	0.059917
	hsa00534:Heparan sulfate biosynthesis	0.061869
	hsa04270:Vascular smooth muscle contraction	0.069194

	hsa05410:Hypertrophic cardiomyopathy (HCM)	0.085976
	hsa04110:Cell cycle	0.098123

Table S9. Go and Pathway analysis of the apoptosis and tumors migration module of neuroblastoma

**Category**	**Term**	***p* value**
BP(level 4)	GO:0007399~nervous system development	2.22E-13
	GO:0007242~intracellular signaling cascade	1.70E-12
	GO:0007165~signal transduction	9.71E-12
	GO:0046649~lymphocyte activation	1.70E-09
	GO:0048666~neuron development	1.70E-09
	GO:0010646~regulation of cell communication	2.72E-09
	GO:0030182~neuron differentiation	2.83E-09
	GO:0022008~neurogenesis	7.93E-09
	GO:0009966~regulation of signal transduction	1.32E-08
	GO:0000902~cell morphogenesis	2.56E-08
	GO:0030036~actin cytoskeleton organization	3.12E-08
	GO:0032989~cellular component morphogenesis	3.51E-08
	GO:0022603~regulation of anatomical structure morphogenesis	4.63E-08
	GO:0022604~regulation of cell morphogenesis	1.02E-07
	GO:0048522~positive regulation of cellular process	1.36E-07
	GO:0051270~regulation of cell motion	1.37E-07
	GO:0000904~cell morphogenesis involved in differentiation	1.58E-07
	GO:0048812~neuron projection morphogenesis	1.98E-07
	GO:0002697~regulation of immune effector process	2.17E-07
	GO:0048468~cell development	2.66E-07
	GO:0048513~organ development	2.71E-07
	GO:0007156~homophilic cell adhesion	2.92E-07
	GO:0050865~regulation of cell activation	3.28E-07
	GO:0043068~positive regulation of programmed cell death	5.32E-07
	GO:0030334~regulation of cell migration	5.95E-07
	GO:0010942~positive regulation of cell death	6.76E-07
	GO:0051049~regulation of transport	1.23E-06
	GO:0045595~regulation of cell differentiation	1.29E-06
	GO:0043067~regulation of programmed cell death	1.37E-06
	GO:0051094~positive regulation of developmental process	1.71E-06
	GO:0010941~regulation of cell death	1.80E-06
	GO:0042110~T cell activation	1.87E-06
	GO:0001944~vasculature development	2.30E-06
	GO:0042127~regulation of cell proliferation	2.58E-06
	GO:0006897~endocytosis	3.34E-06
	GO:0016192~vesicle-mediated transport	3.75E-06

GO:0048858~cell projection morphogenesis	3.97E-06
GO:0002694~regulation of leukocyte activation	4.64E-06
GO:0032990~cell part morphogenesis	4.82E-06
GO:0048514~blood vessel morphogenesis	7.71E-06
GO:0050776~regulation of immune response	8.51E-06
GO:0019226~transmission of nerve impulse	8.67E-06
GO:0051249~regulation of lymphocyte activation	1.14E-05
GO:0006796~phosphate metabolic process	1.17E-05
GO:0006869~lipid transport	1.53E-05
GO:0051174~regulation of phosphorus metabolic process	1.55E-05
GO:0006954~inflammatory response	1.65E-05
GO:0045597~positive regulation of cell differentiation	2.67E-05
GO:0001501~skeletal system development	2.69E-05
GO:0051338~regulation of transferase activity	5.56E-05
GO:0048523~negative regulation of cellular process	6.63E-05
GO:0048870~cell motility	7.06E-05
GO:0060341~regulation of cellular localization	8.85E-05
GO:0060627~regulation of vesicle-mediated transport	1.00E-04
GO:0030155~regulation of cell adhesion	1.01E-04
GO:0008360~regulation of cell shape	1.09E-04
GO:0001817~regulation of cytokine production	1.19E-04
GO:0016477~cell migration	1.22E-04
GO:0007268~synaptic transmission	1.44E-04
GO:0032940~secretion by cell	1.46E-04
GO:0008285~negative regulation of cell proliferation	1.51E-04
GO:0030705~cytoskeleton-dependent intracellular transport	1.98E-04
GO:0002703~regulation of leukocyte mediated immunity	2.45E-04
GO:0002683~negative regulation of immune system process	2.72E-04
GO:0001525~angiogenesis	2.78E-04
GO:0046903~secretion	3.07E-04
GO:0051050~positive regulation of transport	3.12E-04
GO:0048534~hemopoietic or lymphoid organ development	3.59E-04
GO:0001558~regulation of cell growth	3.60E-04
GO:0040017~positive regulation of locomotion	3.69E-04
GO:0051272~positive regulation of cell motion	3.69E-04
GO:0030335~positive regulation of cell migration	3.74E-04
GO:0030097~hemopoiesis	4.33E-04
GO:0006915~apoptosis	4.41E-04
GO:0042060~wound healing	4.65E-04
GO:0010769~regulation of cell morphogenesis involved in differentiation	5.01E-04
GO:0045471~response to ethanol	5.15E-04

	GO:0051046~regulation of secretion	5.22E-04
	GO:0032880~regulation of protein localization	5.46E-04
	GO:0051093~negative regulation of developmental process	5.62E-04
	GO:0031328~positive regulation of cellular biosynthetic process	6.01E-04
	GO:0006909~phagocytosis	6.43E-04
	GO:0050866~negative regulation of cell activation	7.01E-04
	GO:0050777~negative regulation of immune response	7.27E-04
	GO:0044057~regulation of system process	7.41E-04
	GO:0010975~regulation of neuron projection development	7.45E-04
	GO:0051101~regulation of DNA binding	7.69E-04
	GO:0009891~positive regulation of biosynthetic process	7.87E-04
	GO:0009968~negative regulation of signal transduction	8.06E-04
	GO:0007157~heterophilic cell adhesion	9.31E-04
	GO:0002520~immune system development	0.001087
	GO:0045619~regulation of lymphocyte differentiation	0.001108
	GO:0031347~regulation of defense response	0.001121
	GO:0048660~regulation of smooth muscle cell proliferation	0.001151
	GO:0017157~regulation of exocytosis	0.001217
	GO:0010648~negative regulation of cell communication	0.001545
	GO:0070201~regulation of establishment of protein localization	0.001598
	GO:0002521~leukocyte differentiation	0.001646
	GO:0007411~axon guidance	0.001728
	GO:0040013~negative regulation of locomotion	0.001875
	GO:0045637~regulation of myeloid cell differentiation	0.001877
	GO:0051129~negative regulation of cellular component organization	0.001908
	GO:0032943~mononuclear cell proliferation	0.002045
	GO:0031344~regulation of cell projection organization	0.002047
	GO:0007416~synaptogenesis	0.002262
	GO:0051223~regulation of protein transport	0.002387
	GO:0045596~negative regulation of cell differentiation	0.002481
	GO:0048585~negative regulation of response to stimulus	0.002595
	GO:0006811~ion transport	0.002686
	GO:0010557~positive regulation of macromolecule biosynthetic process	0.002759
	GO:0002700~regulation of production of molecular mediator of immune response	0.002805
	GO:0051250~negative regulation of lymphocyte activation	0.00281
	GO:0045088~regulation of innate immune response	0.00281
	GO:0050772~positive regulation of axonogenesis	0.003
	GO:0050867~positive regulation of cell activation	0.003134

	GO:0008284~positive regulation of cell proliferation	0.003213
	GO:0051254~positive regulation of RNA metabolic process	0.003226
	GO:0051251~positive regulation of lymphocyte activation	0.003434
	GO:0019216~regulation of lipid metabolic process	0.003608
	GO:0022405~hair cycle process	0.00361
	GO:0046651~lymphocyte proliferation	0.00361
	GO:0042551~neuron maturation	0.003622
	GO:0007269~neurotransmitter secretion	0.004055
	GO:0030098~lymphocyte differentiation	0.00406
	GO:0045087~innate immune response	0.004107
	GO:0006812~cation transport	0.004153
	GO:0007517~muscle organ development	0.004284
	GO:0048646~anatomical structure formation involved in morphogenesis	0.004376
	GO:0007162~negative regulation of cell adhesion	0.004593
	GO:0048469~cell maturation	0.004681
	GO:0051240~positive regulation of multicellular organismal process	0.004712
	GO:0051047~positive regulation of secretion	0.004721
	GO:0002695~negative regulation of leukocyte activation	0.00523
	GO:0050900~leukocyte migration	0.00523
	GO:0030336~negative regulation of cell migration	0.00523
	GO:0016358~dendrite development	0.005305
	GO:0048878~chemical homeostasis	0.005432
	GO:0048661~positive regulation of smooth muscle cell proliferation	0.005581
	GO:0009887~organ morphogenesis	0.005665
	GO:0014910~regulation of smooth muscle cell migration	0.005831
	GO:0051222~positive regulation of protein transport	0.005986
	GO:0080134~regulation of response to stress	0.006052
	GO:0002696~positive regulation of leukocyte activation	0.006166
	GO:0006873~cellular ion homeostasis	0.00639
	GO:0002460~adaptive immune response based on somatic recombination of immune receptors built from immunoglobulin superfamily domains	0.006508
	GO:0051271~negative regulation of cell motion	0.006762
	GO:0006461~protein complex assembly	0.007019
	GO:0051259~protein oligomerization	0.007142
	GO:0045577~regulation of B cell differentiation	0.007183
	GO:0045446~endothelial cell differentiation	0.007183
	GO:0046890~regulation of lipid biosynthetic process	0.007205
	GO:0001936~regulation of endothelial cell proliferation	0.00732
	GO:0051173~positive regulation of nitrogen compound	0.007355

metabolic process	
GO:0007159~leukocyte adhesion	0.0077
GO:0060113~inner ear receptor cell differentiation	0.007898
GO:0050714~positive regulation of protein secretion	0.007948
GO:0010720~positive regulation of cell development	0.008405
GO:0010604~positive regulation of macromolecule metabolic process	0.008599
GO:0051494~negative regulation of cytoskeleton organization	0.008604
GO:0042035~regulation of cytokine biosynthetic process	0.008675
GO:0001763~morphogenesis of a branching structure	0.008675
GO:0045935~positive regulation of nucleobase, nucleoside, nucleotide and nucleic acid metabolic process	0.00875
GO:0043412~biopolymer modification	0.008966
GO:0070663~regulation of leukocyte proliferation	0.009027
GO:0006887~exocytosis	0.010197
GO:0002764~immune response-regulating signal transduction	0.010331
GO:0002819~regulation of adaptive immune response	0.010331
GO:0001910~regulation of leukocyte mediated cytotoxicity	0.010653
GO:0010594~regulation of endothelial cell migration	0.010653
GO:0016049~cell growth	0.010849
GO:0002684~positive regulation of immune system process	0.011147
GO:0051969~regulation of transmission of nerve impulse	0.011311
GO:0001822~kidney development	0.011963
GO:0048535~lymph node development	0.011964
GO:0019229~regulation of vasoconstriction	0.012036
GO:0031644~regulation of neurological system process	0.012194
GO:0010563~negative regulation of phosphorus metabolic process	0.013194
GO:0010647~positive regulation of cell communication	0.013253
GO:0009893~positive regulation of metabolic process	0.013512
GO:0030308~negative regulation of cell growth	0.013682
GO:0010628~positive regulation of gene expression	0.013831
GO:0032365~intracellular lipid transport	0.014221
GO:0006935~chemotaxis	0.014395
GO:0042330~taxis	0.014395
GO:0022407~regulation of cell-cell adhesion	0.014787
GO:0007050~cell cycle arrest	0.015204
GO:0031349~positive regulation of defense response	0.015577
GO:0006836~neurotransmitter transport	0.015705
GO:0007166~cell surface receptor linked signal transduction	0.016659

	GO:0032680~regulation of tumor necrosis factor production	0.016687
	GO:0009306~protein secretion	0.016687
	GO:0060284~regulation of cell development	0.01695
	GO:0031325~positive regulation of cellular metabolic process	0.017179
	GO:0051100~negative regulation of binding	0.017195
	GO:0007160~cell-matrix adhesion	0.017804
	GO:0046907~intracellular transport	0.01828
	GO:0051493~regulation of cytoskeleton organization	0.01842
	GO:0009967~positive regulation of signal transduction	0.018743
	GO:0007422~peripheral nervous system development	0.018764
	GO:0001655~urogenital system development	0.018827
	GO:0002820~negative regulation of adaptive immune response	0.019134
	GO:0030041~actin filament polymerization	0.019134
	GO:0009888~tissue development	0.020062
	GO:0007612~learning	0.020128
	GO:0001570~vasculogenesis	0.02037
	GO:0043242~negative regulation of protein complex disassembly	0.02037
	GO:0048871~multicellular organismal homeostasis	0.020396
	GO:0003001~generation of a signal involved in cell-cell signaling	0.020396
	GO:0048754~branching morphogenesis of a tube	0.020432
	GO:0002758~innate immune response-activating signal transduction	0.020607
	GO:0048483~autonomic nervous system development	0.020607
	GO:0031069~hair follicle morphogenesis	0.020607
	GO:0016079~synaptic vesicle exocytosis	0.02073
	GO:0002274~myeloid leukocyte activation	0.021577
	GO:0048584~positive regulation of response to stimulus	0.02212
	GO:0051130~positive regulation of cellular component organization	0.022143
	GO:0051960~regulation of nervous system development	0.022293
	GO:0015918~sterol transport	0.023019
	GO:0002443~leukocyte mediated immunity	0.023115
	GO:0031345~negative regulation of cell projection organization	0.023222
	GO:0008643~carbohydrate transport	0.023427
	GO:0042113~B cell activation	0.023569
	GO:0001649~osteoblast differentiation	0.024561
	GO:0030857~negative regulation of epithelial cell differentiation	0.02494

	GO:0006576~biogenic amine metabolic process	0.025132
	GO:0006650~glycerophospholipid metabolic process	0.02529
	GO:0048638~regulation of developmental growth	0.025667
	GO:0031346~positive regulation of cell projection organization	0.025667
	GO:0015914~phospholipid transport	0.025941
	GO:0046486~glycerolipid metabolic process	0.027933
	GO:0042552~myelination	0.027936
	GO:0051051~negative regulation of transport	0.027965
	GO:0030035~microspike assembly	0.028858
	GO:0002698~negative regulation of immune effector process	0.028858
	GO:0010970~microtubule-based transport	0.029113
	GO:0001562~response to protozoan	0.030118
	GO:0043066~negative regulation of apoptosis	0.030256
	GO:0051336~regulation of hydrolase activity	0.030571
	GO:0050880~regulation of blood vessel size	0.030881
	GO:0003018~vascular process in circulatory system	0.031169
	GO:0050708~regulation of protein secretion	0.031169
	GO:0045792~negative regulation of cell size	0.031363
	GO:0010827~regulation of glucose transport	0.031734
	GO:0048730~epidermis morphogenesis	0.032465
	GO:0006936~muscle contraction	0.032763
	GO:0051241~negative regulation of multicellular organismal process	0.032975
	GO:0032101~regulation of response to external stimulus	0.034276
	GO:0010562~positive regulation of phosphorus metabolic process	0.034885
	GO:0031099~regeneration	0.035426
	GO:0007163~establishment or maintenance of cell polarity	0.03551
	GO:0046889~positive regulation of lipid biosynthetic process	0.035826
	GO:0030101~natural killer cell activation	0.035826
	GO:0001569~patterning of blood vessels	0.035826
	GO:0008344~adult locomotory behavior	0.035868
	GO:0050707~regulation of cytokine secretion	0.035974
	GO:0032870~cellular response to hormone stimulus	0.03805
	GO:0045621~positive regulation of lymphocyte differentiation	0.038379
	GO:0050767~regulation of neurogenesis	0.038688
	GO:0032655~regulation of interleukin-12 production	0.038828
	GO:0002474~antigen processing and presentation of peptide antigen via MHC class I	0.038828
	GO:0043069~negative regulation of programmed cell death	0.03959

GO:0048568~embryonic organ development	0.040146
GO:0045191~regulation of isotype switching	0.040655
GO:0060548~negative regulation of cell death	0.0417
GO:0019725~cellular homeostasis	0.044383
GO:0032386~regulation of intracellular transport	0.045391
GO:0043542~endothelial cell migration	0.045645
GO:0032675~regulation of interleukin-6 production	0.045924
GO:0002366~leukocyte activation during immune response	0.045924
GO:0043085~positive regulation of catalytic activity	0.046455
GO:0030100~regulation of endocytosis	0.046997
GO:0045834~positive regulation of lipid metabolic process	0.047694
GO:0045089~positive regulation of innate immune response	0.047694
GO:0045580~regulation of T cell differentiation	0.047788
GO:0060537~muscle tissue development	0.048904
GO:0008015~blood circulation	0.049505
GO:0008016~regulation of heart contraction	0.049765
GO:0010464~regulation of mesenchymal cell proliferation	0.050739
GO:0030501~positive regulation of bone mineralization	0.050739
GO:0001937~negative regulation of endothelial cell proliferation	0.050739
GO:0045646~regulation of erythrocyte differentiation	0.050739
GO:0045055~regulated secretory pathway	0.050739
GO:0007015~actin filament organization	0.051057
GO:0001503~ossification	0.052833
GO:0009755~hormone-mediated signaling	0.054911
GO:0030318~melanocyte differentiation	0.055294
GO:0014070~response to organic cyclic substance	0.055443
GO:0043271~negative regulation of ion transport	0.057029
GO:0050715~positive regulation of cytokine secretion	0.057029
GO:0001504~neurotransmitter uptake	0.057696
GO:0048875~chemical homeostasis within a tissue	0.057696
GO:0007413~axonal fasciculation	0.057696
GO:0035239~tube morphogenesis	0.057929
GO:0032271~regulation of protein polymerization	0.058776
GO:0048562~embryonic organ morphogenesis	0.060294
GO:0016197~endosome transport	0.061589
GO:0045639~positive regulation of myeloid cell differentiation	0.062917
GO:0048489~synaptic vesicle transport	0.062917
GO:0010721~negative regulation of cell development	0.063562
GO:0001819~positive regulation of cytokine production	0.063791
GO:0008154~actin polymerization or depolymerization	0.064646
GO:0046626~regulation of insulin receptor signaling	0.064646

pathway	
GO:0034103~regulation of tissue remodeling	0.064646
GO:0070169~positive regulation of biomineral formation	0.064646
GO:0048536~spleen development	0.064646
GO:0006644~phospholipid metabolic process	0.064865
GO:0001666~response to hypoxia	0.065251
GO:0070296~sarcoplasmic reticulum calcium ion transport	0.066167
GO:0050701~interleukin-1 secretion	0.066167
GO:0032103~positive regulation of response to external stimulus	0.067749
GO:0006904~vesicle docking during exocytosis	0.070018
GO:0048732~gland development	0.070487
GO:0003002~regionalization	0.07057
GO:0048729~tissue morphogenesis	0.070751
GO:0043279~response to alkaloid	0.071234
GO:0050931~pigment cell differentiation	0.072576
GO:0046627~negative regulation of insulin receptor signaling pathway	0.072576
GO:0001578~microtubule bundle formation	0.072576
GO:0046850~regulation of bone remodeling	0.072576
GO:0031290~retinal ganglion cell axon guidance	0.072576
GO:0045124~regulation of bone resorption	0.072576
GO:0001938~positive regulation of endothelial cell proliferation	0.072576
GO:0030856~regulation of epithelial cell differentiation	0.072892
GO:0002449~lymphocyte mediated immunity	0.073421
GO:0031333~negative regulation of protein complex assembly	0.074143
GO:0002429~immune response-activating cell surface receptor signaling pathway	0.074309
GO:0051781~positive regulation of cell division	0.074309
GO:0050727~regulation of inflammatory response	0.078643
GO:0048640~negative regulation of developmental growth	0.079732
GO:0051253~negative regulation of RNA metabolic process	0.080296
GO:0051181~cofactor transport	0.080561
GO:0050678~regulation of epithelial cell proliferation	0.081575
GO:0035095~behavioral response to nicotine	0.082573
GO:0015879~carnitine transport	0.082573
GO:0060073~micturition	0.082573
GO:0007130~synaptonemal complex assembly	0.082573
GO:0001885~endothelial cell development	0.082573
GO:0010639~negative regulation of organelle organization	0.083451
GO:0033273~response to vitamin	0.084538

	GO:0050680~negative regulation of epithelial cell proliferation	0.084622
	GO:0050771~negative regulation of axonogenesis	0.084622
	GO:0051224~negative regulation of protein transport	0.085762
	GO:0003073~regulation of systemic arterial blood pressure	0.086522
	GO:0042692~muscle cell differentiation	0.086748
	GO:0045926~negative regulation of growth	0.087633
	GO:0006937~regulation of muscle contraction	0.090295
	GO:0070665~positive regulation of leukocyte proliferation	0.090426
	GO:0009408~response to heat	0.090426
	GO:0030032~lamellipodium assembly	0.092448
	GO:0007026~negative regulation of microtubule depolymerization	0.092448
	GO:0031114~regulation of microtubule depolymerization	0.092448
	GO:0043244~regulation of protein complex disassembly	0.093252
	GO:0032956~regulation of actin cytoskeleton organization	0.096045
	GO:0050778~positive regulation of immune response	0.09654
	GO:0002761~regulation of myeloid leukocyte differentiation	0.098225
	GO:0046928~regulation of neurotransmitter secretion	0.098455
	GO:0043029~T cell homeostasis	0.098455
	GO:0002064~epithelial cell development	0.098455
	GO:0060249~anatomical structure homeostasis	0.09922
MF(level 4)	GO:0003779~actin binding	2.86E-09
	GO:0005085~guanyl-nucleotide exchange factor activity	7.40E-08
	GO:0005083~small GTPase regulator activity	8.66E-06
	GO:0051020~GTPase binding	2.35E-05
	GO:0016773~phosphotransferase activity, alcohol group as acceptor	9.85E-05
	GO:0005070~SH3/SH2 adaptor activity	1.07E-04
	GO:0005096~GTPase activator activity	1.10E-04
	GO:0016301~kinase activity	1.23E-04
	GO:0005158~insulin receptor binding	6.93E-04
	GO:0017124~SH3 domain binding	8.61E-04
	GO:0042803~protein homodimerization activity	0.001221
	GO:0019900~kinase binding	0.002005
	GO:0005099~Ras GTPase activator activity	0.003879
	GO:0030165~PDZ domain binding	0.005247
	GO:0015631~tubulin binding	0.007204
	GO:0008603~cAMP-dependent protein kinase regulator activity	0.008163
	GO:0001664~G-protein-coupled receptor binding	0.008413
	GO:0042169~SH2 domain binding	0.009182
	GO:0005548~phospholipid transporter activity	0.011868

	GO:0005520~insulin-like growth factor binding	0.012341
	GO:0042578~phosphoric ester hydrolase activity	0.01433
	GO:0004896~cytokine receptor activity	0.023772
	GO:0035091~phosphoinositide binding	0.025158
	GO:0015026~coreceptor activity	0.030842
	GO:0005159~insulin-like growth factor receptor binding	0.031362
	GO:0015075~ion transmembrane transporter activity	0.038027
	GO:0043120~tumor necrosis factor binding	0.042494
	GO:0019905~syntaxin binding	0.049787
	GO:0032555~purine ribonucleotide binding	0.055969
	GO:0015267~channel activity	0.061235
	GO:0050431~transforming growth factor beta binding	0.062032
	GO:0046982~protein heterodimerization activity	0.064685
	GO:0005178~integrin binding	0.081062
	GO:0030554~adenyl nucleotide binding	0.089522
KEGG Pathway	hsa04514:Cell adhesion molecules (CAMs)	2.27E-08
	hsa04666:Fc gamma R-mediated phagocytosis	3.18E-05
	hsa04062:Chemokine signaling pathway	4.75E-04
	hsa04060:Cytokine-cytokine receptor interaction	5.69E-04
	hsa04210:Apoptosis	5.88E-04
	hsa05200:Pathways in cancer	5.90E-04
	hsa04810:Regulation of actin cytoskeleton	7.58E-04
	hsa05412:Arrhythmogenic right ventricular cardiomyopathy (ARVC)	8.87E-04
	hsa05414:Dilated cardiomyopathy	0.001515
	hsa05410:Hypertrophic cardiomyopathy (HCM)	0.002161
	hsa04510:Focal adhesion	0.002581
	hsa04070:Phosphatidylinositol signaling system	0.003367
	hsa04010:MAPK signaling pathway	0.004235
	hsa04360:Axon guidance	0.005727
	hsa04512:ECM-receptor interaction	0.008361
	hsa04650:Natural killer cell mediated cytotoxicity	0.009311
	hsa05221:Acute myeloid leukemia	0.010913
	hsa04520:Adherens junction	0.011991
	hsa04940:Type I diabetes mellitus	0.015468
	hsa04144:Endocytosis	0.016353
	hsa05215:Prostate cancer	0.017018
	hsa04930:Type II diabetes mellitus	0.017286
	hsa04916:Melanogenesis	0.01763
	hsa05416:Viral myocarditis	0.019995
	hsa04640:Hematopoietic cell lineage	0.021669
	hsa05330:Allograft rejection	0.027463
	hsa05222:Small cell lung cancer	0.031185

	hsa04670:Leukocyte transendothelial migration	0.046411
	hsa05332:Graft-versus-host disease	0.047934
	hsa04630:Jak-STAT signaling pathway	0.048448
	hsa04672:Intestinal immune network for IgA production	0.052417
	hsa04540:Gap junction	0.05502
	hsa05217:Basal cell carcinoma	0.061613
	hsa04620:Toll-like receptor signaling pathway	0.065454
	hsa00533:Keratan sulfate biosynthesis	0.067972
	hsa04270:Vascular smooth muscle contraction	0.069135
	hsa04020:Calcium signaling pathway	0.070497
	hsa04660:T cell receptor signaling pathway	0.07701
	hsa04621:NOD-like receptor signaling pathway	0.079353
	hsa04912:GnRH signaling pathway	0.080169
	hsa04720:Long-term potentiation	0.088108
	hsa05120:Epithelial cell signaling in Helicobacter pylori infection	0.088108
	hsa05210:Colorectal cancer	0.09311

Table S10. Go and Pathway analysis of the nervous system development module of neuroblastoma

**Category**	**Term**	***p* value**
BP(level 4)	GO:0048513~organ development	1.26E-08
	GO:0030855~epithelial cell differentiation	3.87E-06
	GO:0007398~ectoderm development	8.88E-06
	GO:0030216~keratinocyte differentiation	2.33E-05
	GO:0008544~epidermis development	3.69E-05
	GO:0007166~cell surface receptor linked signal transduction	5.34E-05
	GO:0009913~epidermal cell differentiation	6.89E-05
	GO:0009888~tissue development	1.71E-04
	GO:0006811~ion transport	2.12E-04
	GO:0060429~epithelium development	2.72E-04
	GO:0007165~signal transduction	5.61E-04
	GO:0048878~chemical homeostasis	0.001713
	GO:0007517~muscle organ development	0.001903
	GO:0007620~copulation	0.002279
	GO:0001501~skeletal system development	0.002695
	GO:0007320~insemination	0.00452
	GO:0050890~cognition	0.004622
	GO:0007399~nervous system development	0.005816
	GO:0006936~muscle contraction	0.006727
	GO:0006873~cellular ion homeostasis	0.00673
	GO:0019226~transmission of nerve impulse	0.006813
	GO:0006812~cation transport	0.008323

GO:0035112~genitalia morphogenesis	0.008562
GO:0042060~wound healing	0.009007
GO:0046942~carboxylic acid transport	0.009448
GO:0015849~organic acid transport	0.010105
GO:0048871~multicellular organismal homeostasis	0.010856
GO:0048704~embryonic skeletal system morphogenesis	0.0129
GO:0031279~regulation of cyclase activity	0.014711
GO:0051093~negative regulation of developmental process	0.014835
GO:0006820~anion transport	0.015074
GO:0032846~positive regulation of homeostatic process	0.015836
GO:0044057~regulation of system process	0.015885
GO:0048806~genitalia development	0.016348
GO:0007268~synaptic transmission	0.016715
GO:0051339~regulation of lyase activity	0.017178
GO:0007338~single fertilization	0.017744
GO:0046903~secretion	0.018192
GO:0051591~response to cAMP	0.020418
GO:0042110~T cell activation	0.021304
GO:0048568~embryonic organ development	0.021692
GO:0003079~positive regulation of natriuresis	0.024585
GO:0032849~positive regulation of cellular pH reduction	0.024585
GO:0002118~aggressive behavior	0.024585
GO:0060249~anatomical structure homeostasis	0.024709
GO:0007599~hemostasis	0.02832
GO:0007156~homophilic cell adhesion	0.029092
GO:0042692~muscle cell differentiation	0.031673
GO:0070665~positive regulation of leukocyte proliferation	0.032098
GO:0045596~negative regulation of cell differentiation	0.032649
GO:0048562~embryonic organ morphogenesis	0.032733
GO:0048545~response to steroid hormone stimulus	0.032884
GO:0051222~positive regulation of protein transport	0.033968
GO:0048732~gland development	0.036695
GO:0051969~regulation of transmission of nerve impulse	0.037341
GO:0032847~regulation of cellular pH reduction	0.03917
GO:0042713~sperm ejaculation	0.03917
GO:0043288~apocarotenoid metabolic process	0.03917
GO:0042574~retinal metabolic process	0.03917
GO:0002070~epithelial cell maturation	0.03917
GO:0051024~positive regulation of immunoglobulin secretion	0.03917
GO:0007596~blood coagulation	0.039884
GO:0007340~acrosome reaction	0.041856
GO:0007417~central nervous system development	0.042141
GO:0009583~detection of light stimulus	0.043703

	GO:0051050~positive regulation of transport	0.044109
	GO:0019725~cellular homeostasis	0.044279
	GO:0046649~lymphocyte activation	0.045237
	GO:0002703~regulation of leukocyte mediated immunity	0.049815
	GO:0007420~brain development	0.049829
	GO:0031644~regulation of neurological system process	0.050772
	GO:0048468~cell development	0.050977
	GO:0042445~hormone metabolic process	0.051117
	GO:0014048~regulation of glutamate secretion	0.051776
	GO:0070663~regulation of leukocyte proliferation	0.053724
	GO:0046661~male sex differentiation	0.054288
	GO:0014049~positive regulation of glutamate secretion	0.05618
	GO:0010669~epithelial structure maintenance	0.05618
	GO:0030277~maintenance of gastrointestinal epithelium	0.05618
	GO:0051047~positive regulation of secretion	0.060836
	GO:0015807~L-amino acid transport	0.064104
	GO:0043085~positive regulation of catalytic activity	0.064697
	GO:0021537~telencephalon development	0.067826
	GO:0046546~development of primary male sexual characteristics	0.067826
	GO:0008016~regulation of heart contraction	0.071484
	GO:0048705~skeletal system morphogenesis	0.071707
	GO:0007160~cell-matrix adhesion	0.073764
	GO:0060425~lung morphogenesis	0.075228
	GO:0042127~regulation of cell proliferation	0.076908
	GO:0002819~regulation of adaptive immune response	0.079036
	GO:0007548~sex differentiation	0.081382
	GO:0019827~stem cell maintenance	0.082522
	GO:0006801~superoxide metabolic process	0.082522
	GO:0001503~ossification	0.083758
	GO:0008202~steroid metabolic process	0.084448
	GO:0022008~neurogenesis	0.088185
	GO:0048598~embryonic morphogenesis	0.089384
	GO:0021782~glial cell development	0.092602
	GO:0048864~stem cell development	0.092602
	GO:0051960~regulation of nervous system development	0.09334
	GO:0009887~organ morphogenesis	0.093879
	GO:0045060~negative thymic T cell selection	0.095963
	GO:0021772~olfactory bulb development	0.095963
	GO:0048266~behavioral response to pain	0.095963
	GO:0003078~regulation of natriuresis	0.095963
	GO:0051971~positive regulation of transmission of nerve impulse	0.098798
MF(level 4)	GO:0004888~transmembrane receptor activity	4.91E-06

	GO:0005125~cytokine activity	1.78E-04
	GO:0015267~channel activity	4.64E-04
	GO:0001653~peptide receptor activity	8.03E-04
	GO:0008528~peptide receptor activity, G-protein coupled	8.03E-04
	GO:0015075~ion transmembrane transporter activity	0.003978
	GO:0030594~neurotransmitter receptor activity	0.013579
	GO:0005085~guanyl-nucleotide exchange factor activity	0.033751
	GO:0003700~transcription factor activity	0.034231
	GO:0048019~receptor antagonist activity	0.035863
	GO:0050664~oxidoreductase activity, acting on NADH or NADPH, with oxygen as acceptor	0.035863
	GO:0031013~troponin I binding	0.041591
	GO:0016594~glycine binding	0.045417
	GO:0015291~secondary active transmembrane transporter activity	0.049921
	GO:0016712~oxidoreductase activity, acting on paired donors, with incorporation or reduction of molecular oxygen, reduced flavin or flavoprotein as one donor, and incorporation of one atom of oxygen	0.052899
	GO:0030545~receptor regulator activity	0.052899
	GO:0008188~neuropeptide receptor activity	0.055328
	GO:0005523~tropomyosin binding	0.05609
	GO:0043565~sequence-specific DNA binding	0.063834
	GO:0005179~hormone activity	0.069675
	GO:0070279~vitamin B6 binding	0.0774
	GO:0030170~pyridoxal phosphate binding	0.0774
	GO:0016836~hydro-lyase activity	0.080897
	GO:0008083~growth factor activity	0.091328
KEGG Pathway	hsa04080:Neuroactive ligand-receptor interaction	1.11E-05
	hsa04060:Cytokine-cytokine receptor interaction	0.001081
	hsa04742:Taste transduction	0.006681
	hsa05414:Dilated cardiomyopathy	0.018251
	hsa04020:Calcium signaling pathway	0.02422
	hsa05410:Hypertrophic cardiomyopathy (HCM)	0.024561
	hsa04630:Jak-STAT signaling pathway	0.05205
	hsa05412:Arrhythmogenic right ventricular cardiomyopathy (ARVC)	0.064227
	hsa04270:Vascular smooth muscle contraction	0.068113
	hsa00590:Arachidonic acid metabolism	0.076595
	hsa00120:Primary bile acid biosynthesis	0.086163

Table S11. Go and Pathway analysis of the apoptosis and cell death regulation module of glioblastoma_seq

Category	Term	*p* value
BP(level 4)	GO:0006954~inflammatory response	8.02E-25
	GO:0006935~chemotaxis	7.48E-13
	GO:0042330~taxis	7.48E-13
	GO:0002684~positive regulation of immune system process	1.02E-09
	GO:0048522~positive regulation of cellular process	2.00E-08
	GO:0050776~regulation of immune response	5.42E-08
	GO:0002460~adaptive immune response based on somatic recombination of immune receptors built from immunoglobulin superfamily domains	9.47E-08
	GO:0042127~regulation of cell proliferation	1.14E-07
	GO:0043067~regulation of programmed cell death	5.06E-07
	GO:0048584~positive regulation of response to stimulus	5.12E-07
	GO:0010941~regulation of cell death	5.88E-07
	GO:0002253~activation of immune response	8.62E-07
	GO:0016052~carbohydrate catabolic process	9.14E-07
	GO:0043066~negative regulation of apoptosis	1.11E-06
	GO:0043069~negative regulation of programmed cell death	1.71E-06
	GO:0060548~negative regulation of cell death	1.87E-06
	GO:0002449~lymphocyte mediated immunity	1.99E-06
	GO:0045087~innate immune response	2.27E-06
	GO:0048523~negative regulation of cellular process	2.29E-06
	GO:0002443~leukocyte mediated immunity	2.99E-06
	GO:0048545~response to steroid hormone stimulus	3.68E-06
	GO:0006956~complement activation	5.75E-06
	GO:0050778~positive regulation of immune response	6.05E-06
	GO:0001817~regulation of cytokine production	7.75E-06
	GO:0042060~wound healing	8.92E-06
	GO:0050865~regulation of cell activation	1.06E-05
	GO:0001666~response to hypoxia	1.28E-05
	GO:0006096~glycolysis	2.25E-05
	GO:0019725~cellular homeostasis	2.54E-05
	GO:0002694~regulation of leukocyte activation	2.81E-05
	GO:0006915~apoptosis	4.28E-05
	GO:0051240~positive regulation of multicellular organismal process	4.48E-05
	GO:0002455~humoral immune response mediated by circulating immunoglobulin	4.67E-05
	GO:0005976~polysaccharide metabolic process	5.00E-05
	GO:0001819~positive regulation of cytokine production	8.01E-05
	GO:0001944~vasculature development	8.16E-05
	GO:0032101~regulation of response to external stimulus	9.17E-05
	GO:0008284~positive regulation of cell proliferation	9.53E-05
	GO:0002700~regulation of production of molecular	1.29E-04

mediator of immune response	
GO:0002237~response to molecule of bacterial origin	1.46E-04
GO:0006958~complement activation, classical pathway	1.57E-04
GO:0048514~blood vessel morphogenesis	1.60E-04
GO:0009617~response to bacterium	1.74E-04
GO:0001525~angiogenesis	1.92E-04
GO:0051604~protein maturation	2.04E-04
GO:0032680~regulation of tumor necrosis factor production	2.77E-04
GO:0051094~positive regulation of developmental process	3.06E-04
GO:0007165~signal transduction	3.12E-04
GO:0001936~regulation of endothelial cell proliferation	3.60E-04
GO:0019318~hexose metabolic process	3.70E-04
GO:0045595~regulation of cell differentiation	3.85E-04
GO:0060326~cell chemotaxis	3.90E-04
GO:0005996~monosaccharide metabolic process	4.01E-04
GO:0044275~cellular carbohydrate catabolic process	4.06E-04
GO:0032496~response to lipopolysaccharide	4.16E-04
GO:0050867~positive regulation of cell activation	4.33E-04
GO:0010883~regulation of lipid storage	5.27E-04
GO:0016477~cell migration	5.39E-04
GO:0045765~regulation of angiogenesis	5.58E-04
GO:0046365~monosaccharide catabolic process	5.64E-04
GO:0031667~response to nutrient levels	5.64E-04
GO:0000272~polysaccharide catabolic process	6.81E-04
GO:0006022~aminoglycan metabolic process	7.63E-04
GO:0030199~collagen fibril organization	8.80E-04
GO:0002683~negative regulation of immune system process	9.48E-04
GO:0002697~regulation of immune effector process	0.001034
GO:0048870~cell motility	0.001053
GO:0010647~positive regulation of cell communication	0.001112
GO:0030595~leukocyte chemotaxis	0.001145
GO:0006873~cellular ion homeostasis	0.001266
GO:0009620~response to fungus	0.00129
GO:0046903~secretion	0.001297
GO:0002703~regulation of leukocyte mediated immunity	0.0014
GO:0001818~negative regulation of cytokine production	0.001404
GO:0044262~cellular carbohydrate metabolic process	0.001412
GO:0007040~lysosome organization	0.00168
GO:0048878~chemical homeostasis	0.001697
GO:0002696~positive regulation of leukocyte activation	0.001787
GO:0080134~regulation of response to stress	0.001802
GO:0009967~positive regulation of signal transduction	0.001804

GO:0007242~intracellular signaling cascade	0.001924
GO:0048513~organ development	0.002008
GO:0001558~regulation of cell growth	0.002066
GO:0001501~skeletal system development	0.002099
GO:0031668~cellular response to extracellular stimulus	0.002155
GO:0007599~hemostasis	0.002196
GO:0006909~phagocytosis	0.002197
GO:0048646~anatomical structure formation involved in morphogenesis	0.002197
GO:0008015~blood circulation	0.002395
GO:0030574~collagen catabolic process	0.002452
GO:0050818~regulation of coagulation	0.002474
GO:0050900~leukocyte migration	0.002566
GO:0051249~regulation of lymphocyte activation	0.002683
GO:0022603~regulation of anatomical structure morphogenesis	0.002863
GO:0046649~lymphocyte activation	0.002951
GO:0006026~aminoglycan catabolic process	0.003223
GO:0032963~collagen metabolic process	0.00341
GO:0001938~positive regulation of endothelial cell proliferation	0.003416
GO:0032760~positive regulation of tumor necrosis factor production	0.003416
GO:0008285~negative regulation of cell proliferation	0.003469
GO:0030193~regulation of blood coagulation	0.003924
GO:0032943~mononuclear cell proliferation	0.004107
GO:0051241~negative regulation of multicellular organismal process	0.004335
GO:0051251~positive regulation of lymphocyte activation	0.004619
GO:0000271~polysaccharide biosynthetic process	0.004807
GO:0016051~carbohydrate biosynthetic process	0.004912
GO:0040017~positive regulation of locomotion	0.005093
GO:0045597~positive regulation of cell differentiation	0.005373
GO:0009615~response to virus	0.005892
GO:0009306~protein secretion	0.006245
GO:0002819~regulation of adaptive immune response	0.007025
GO:0030308~negative regulation of cell growth	0.007034
GO:0007596~blood coagulation	0.007402
GO:0010563~negative regulation of phosphorus metabolic process	0.007469
GO:0051270~regulation of cell motion	0.007745
GO:0045596~negative regulation of cell differentiation	0.008698
GO:0010888~negative regulation of lipid storage	0.008893
GO:0050714~positive regulation of protein secretion	0.008952

GO:0031100~organ regeneration	0.009979
GO:0045766~positive regulation of angiogenesis	0.009979
GO:0046651~lymphocyte proliferation	0.010364
GO:0048247~lymphocyte chemotaxis	0.011058
GO:0034637~cellular carbohydrate biosynthetic process	0.011373
GO:0045191~regulation of isotype switching	0.012109
GO:0030334~regulation of cell migration	0.012297
GO:0051050~positive regulation of transport	0.01287
GO:0045792~negative regulation of cell size	0.013205
GO:0051047~positive regulation of secretion	0.013387
GO:0051174~regulation of phosphorus metabolic process	0.014033
GO:0048872~homeostasis of number of cells	0.014352
GO:0048585~negative regulation of response to stimulus	0.014352
GO:0032675~regulation of interleukin-6 production	0.014404
GO:0045926~negative regulation of growth	0.014484
GO:0051093~negative regulation of developmental process	0.015155
GO:0060341~regulation of cellular localization	0.015173
GO:0014070~response to organic cyclic substance	0.015576
GO:0045088~regulation of innate immune response	0.015995
GO:0051259~protein oligomerization	0.016696
GO:0035313~wound healing, spreading of epidermal cells	0.016736
GO:0043300~regulation of leukocyte degranulation	0.016736
GO:0031349~positive regulation of defense response	0.017014
GO:0031347~regulation of defense response	0.017257
GO:0002274~myeloid leukocyte activation	0.017697
GO:0030099~myeloid cell differentiation	0.018119
GO:0042035~regulation of cytokine biosynthetic process	0.018704
GO:0051049~regulation of transport	0.018739
GO:0051223~regulation of protein transport	0.019581
GO:0002764~immune response-regulating signal transduction	0.020003
GO:0032368~regulation of lipid transport	0.020045
GO:0032663~regulation of interleukin-2 production	0.020045
GO:0048871~multicellular organismal homeostasis	0.02108
GO:0042110~T cell activation	0.022128
GO:0050920~regulation of chemotaxis	0.023357
GO:0009311~oligosaccharide metabolic process	0.023357
GO:0051222~positive regulation of protein transport	0.023698
GO:0048246~macrophage chemotaxis	0.023752
GO:0033003~regulation of mast cell activation	0.023752
GO:0045830~positive regulation of isotype switching	0.023752
GO:0051046~regulation of secretion	0.023784
GO:0010646~regulation of cell communication	0.024176
GO:0032880~regulation of protein localization	0.0245

	GO:0050708~regulation of protein secretion	0.024691
	GO:0009966~regulation of signal transduction	0.024835
	GO:0009267~cellular response to starvation	0.027017
	GO:0051100~negative regulation of binding	0.027305
	GO:0051180~vitamin transport	0.027896
	GO:0030335~positive regulation of cell migration	0.029153
	GO:0033631~cell-cell adhesion mediated by integrin	0.029286
	GO:0045785~positive regulation of cell adhesion	0.030109
	GO:0045637~regulation of myeloid cell differentiation	0.031152
	GO:0070201~regulation of establishment of protein localization	0.03163
	GO:0070555~response to interleukin-1	0.031888
	GO:0019058~viral infectious cycle	0.035704
	GO:0006937~regulation of muscle contraction	0.036956
	GO:0009887~organ morphogenesis	0.037508
	GO:0044264~cellular polysaccharide metabolic process	0.038381
	GO:0043068~positive regulation of programmed cell death	0.038675
	GO:0045055~regulated secretory pathway	0.038684
	GO:0030593~neutrophil chemotaxis	0.038684
	GO:0001937~negative regulation of endothelial cell proliferation	0.038684
	GO:0002285~lymphocyte activation during immune response	0.038684
	GO:0046717~acid secretion	0.038684
	GO:0045454~cell redox homeostasis	0.03971
	GO:0031669~cellular response to nutrient levels	0.039872
	GO:0010942~positive regulation of cell death	0.041141
	GO:0048640~negative regulation of developmental growth	0.041794
	GO:0043330~response to exogenous dsRNA	0.041794
	GO:0010885~regulation of cholesterol storage	0.041794
	GO:0010648~negative regulation of cell communication	0.042075
	GO:0032103~positive regulation of response to external stimulus	0.043324
	GO:0030036~actin cytoskeleton organization	0.043548
	GO:0030155~regulation of cell adhesion	0.043612
	GO:0045428~regulation of nitric oxide biosynthetic process	0.044225
	GO:0002366~leukocyte activation during immune response	0.045358
	GO:0002758~innate immune response-activating signal transduction	0.046241
	GO:0048536~spleen development	0.046241
	GO:0044057~regulation of system process	0.047144
	GO:0048660~regulation of smooth muscle cell proliferation	0.049128
	GO:0050795~regulation of behavior	0.049128
	GO:0045089~positive regulation of innate immune	0.049128

	response	
MF(level4)	GO:0005125~cytokine activity	5.81E-11
	GO:0004866~endopeptidase inhibitor activity	1.61E-06
	GO:0001664~G-protein-coupled receptor binding	1.03E-05
	GO:0005539~glycosaminoglycan binding	2.21E-05
	GO:0048029~monosaccharide binding	1.57E-04
	GO:0004553~hydrolase activity, hydrolyzing O-glycosyl compounds	7.73E-04
	GO:0008484~sulfuric ester hydrolase activity	0.001229
	GO:0016864~intramolecular oxidoreductase activity, transposing S-S bonds	0.003246
	GO:0003779~actin binding	0.003987
	GO:0016862~intramolecular oxidoreductase activity, interconverting keto- and enol-groups	0.005082
	GO:0008236~serine-type peptidase activity	0.005151
	GO:0004896~cytokine receptor activity	0.008642
	GO:0070011~peptidase activity, acting on L-amino acid peptides	0.012458
	GO:0002020~protease binding	0.012665
	GO:0050700~CARD domain binding	0.01268
	GO:0001653~peptide receptor activity	0.014035
	GO:0008528~peptide receptor activity, G-protein coupled	0.014035
	GO:0019956~chemokine binding	0.015135
	GO:0042803~protein homodimerization activity	0.025314
	GO:0043120~tumor necrosis factor binding	0.027081
	GO:0032813~tumor necrosis factor receptor superfamily binding	0.028971
	GO:0016702~oxidoreductase activity, acting on single donors with incorporation of molecular oxygen, incorporation of two atoms of oxygen	0.029109
	GO:0005178~integrin binding	0.035954
	GO:0050699~WW domain binding	0.037359
	GO:0016298~lipase activity	0.043592
	GO:0005544~calcium-dependent phospholipid binding	0.045872
	GO:0016758~transferase activity, transferring hexosyl groups	0.047231
	GO:0048019~receptor antagonist activity	0.047387
KEGG Pathway	hsa04060:Cytokine-cytokine receptor interaction	4.06E-12
	hsa04142:Lysosome	1.82E-07
	hsa04610:Complement and coagulation cascades	4.75E-06
	hsa04062:Chemokine signaling pathway	1.22E-05
	hsa00531:Glycosaminoglycan degradation	3.44E-05
	hsa04670:Leukocyte transendothelial migration	2.09E-04

	hsa00010:Glycolysis / Gluconeogenesis	5.69E-04
	hsa00520:Amino sugar and nucleotide sugar metabolism	0.001196
	hsa04210:Apoptosis	0.001341
	hsa04621:NOD-like receptor signaling pathway	0.00252
	hsa04620:Toll-like receptor signaling pathway	0.002892
	hsa00052:Galactose metabolism	0.006032
	hsa04640:Hematopoietic cell lineage	0.00771
	hsa05120:Epithelial cell signaling in Helicobacter pylori infection	0.014913
	hsa04510:Focal adhesion	0.017546
	hsa00534:Heparan sulfate biosynthesis	0.023184
	hsa04672:Intestinal immune network for IgA production	0.025884
	hsa00051:Fructose and mannose metabolism	0.026675
	hsa00603:Glycosphingolipid biosynthesis	0.029596
	hsa00511:Other glycan degradation	0.046758

Table S12. Go and Pathway analysis of the substance biosynthesis and metabolism module of glioblastoma_seq

**Category**	**Term**	***p* value**
BP (level4)	GO:0001944~vasculature development	1.03E-06
	GO:0048514~blood vessel morphogenesis	3.66E-06
	GO:0048513~organ development	5.65E-06
	GO:0016477~cell migration	1.93E-05
	GO:0048545~response to steroid hormone stimulus	2.36E-05
	GO:0006954~inflammatory response	2.37E-05
	GO:0042330~taxis	5.15E-05
	GO:0006935~chemotaxis	5.15E-05
	GO:0048870~cell motility	5.19E-05
	GO:0044057~regulation of system process	1.42E-04
	GO:0060326~cell chemotaxis	1.99E-04
	GO:0002548~monocyte chemotaxis	2.99E-04
	GO:0030334~regulation of cell migration	3.22E-04
	GO:0050727~regulation of inflammatory response	3.56E-04
	GO:0031347~regulation of defense response	3.93E-04
	GO:0032101~regulation of response to external stimulus	4.47E-04
	GO:0007040~lysosome organization	5.19E-04
	GO:0001525~angiogenesis	5.77E-04
	GO:0001666~response to hypoxia	5.88E-04
	GO:0051270~regulation of cell motion	6.00E-04
	GO:0030595~leukocyte chemotaxis	8.35E-04
	GO:0042060~wound healing	0.001368
	GO:0007160~cell-matrix adhesion	0.001379
	GO:0030155~regulation of cell adhesion	0.002138

GO:0007507~heart development	0.002159
GO:0051047~positive regulation of secretion	0.00233
GO:0044262~cellular carbohydrate metabolic process	0.002339
GO:0050900~leukocyte migration	0.002726
GO:0005996~monosaccharide metabolic process	0.003089
GO:0048660~regulation of smooth muscle cell proliferation	0.003118
GO:0019318~hexose metabolic process	0.00354
GO:0035313~wound healing, spreading of epidermal cells	0.003682
GO:0045785~positive regulation of cell adhesion	0.003777
GO:0002697~regulation of immune effector process	0.003796
GO:0045595~regulation of cell differentiation	0.003802
GO:0031348~negative regulation of defense response	0.003835
GO:0009888~tissue development	0.004243
GO:0048646~anatomical structure formation involved in morphogenesis	0.004674
GO:0042127~regulation of cell proliferation	0.00491
GO:0008016~regulation of heart contraction	0.005347
GO:0032963~collagen metabolic process	0.006038
GO:0016052~carbohydrate catabolic process	0.00676
GO:0050714~positive regulation of protein secretion	0.007398
GO:0005976~polysaccharide metabolic process	0.007729
GO:0040017~positive regulation of locomotion	0.008892
GO:0045667~regulation of osteoblast differentiation	0.009338
GO:0050728~negative regulation of inflammatory response	0.009399
GO:0051174~regulation of phosphorus metabolic process	0.009503
GO:0007520~myoblast fusion	0.009858
GO:0048585~negative regulation of response to stimulus	0.010195
GO:0033631~cell-cell adhesion mediated by integrin	0.010292
GO:0030336~negative regulation of cell migration	0.010309
GO:0002237~response to molecule of bacterial origin	0.010893
GO:0048522~positive regulation of cellular process	0.011272
GO:0003007~heart morphogenesis	0.012273
GO:0022603~regulation of anatomical structure morphogenesis	0.012704
GO:0000768~syncytium formation by plasma membrane fusion	0.012733
GO:0030335~positive regulation of cell migration	0.013467
GO:0080134~regulation of response to stress	0.013654
GO:0040013~negative regulation of locomotion	0.014714
GO:0032940~secretion by cell	0.015904
GO:0016051~carbohydrate biosynthetic process	0.015916
GO:0050776~regulation of immune response	0.016805
GO:0051271~negative regulation of cell motion	0.017357
GO:0030278~regulation of ossification	0.017818

GO:0060537~muscle tissue development	0.01784
GO:0046903~secretion	0.017959
GO:0009617~response to bacterium	0.018336
GO:0032103~positive regulation of response to external stimulus	0.018797
GO:0032102~negative regulation of response to external stimulus	0.01897
GO:0006949~syncytium formation	0.019775
GO:0006812~cation transport	0.02016
GO:0045669~positive regulation of osteoblast differentiation	0.020321
GO:0031667~response to nutrient levels	0.021639
GO:0001818~negative regulation of cytokine production	0.021776
GO:0051241~negative regulation of multicellular organismal process	0.023336
GO:0051272~positive regulation of cell motion	0.023887
GO:0022604~regulation of cell morphogenesis	0.024356
GO:0051046~regulation of secretion	0.026377
GO:0010646~regulation of cell communication	0.028259
GO:0044275~cellular carbohydrate catabolic process	0.028314
GO:0002700~regulation of production of molecular mediator of immune response	0.029315
GO:0001570~vasculogenesis	0.029315
GO:0000272~polysaccharide catabolic process	0.029763
GO:0048878~chemical homeostasis	0.031015
GO:0048247~lymphocyte chemotaxis	0.033078
GO:0030199~collagen fibril organization	0.033397
GO:0006937~regulation of muscle contraction	0.033421
GO:0043043~peptide biosynthetic process	0.033613
GO:0016192~vesicle-mediated transport	0.034632
GO:0050708~regulation of protein secretion	0.036423
GO:0007517~muscle organ development	0.036787
GO:0045768~positive regulation of anti-apoptosis	0.03728
GO:0030593~neutrophil chemotaxis	0.039092
GO:0000302~response to reactive oxygen species	0.040442
GO:0048661~positive regulation of smooth muscle cell proliferation	0.04141
GO:0000271~polysaccharide biosynthetic process	0.041661
GO:0009743~response to carbohydrate stimulus	0.041995
GO:0051702~interaction with symbiont	0.042877
GO:0002703~regulation of leukocyte mediated immunity	0.044971
GO:0050795~regulation of behavior	0.045169
GO:0032496~response to lipopolysaccharide	0.04562
GO:0010769~regulation of cell morphogenesis involved in	0.04562

	differentiation	
	GO:0001936~regulation of endothelial cell proliferation	0.045789
	GO:0007398~ectoderm development	0.045874
	GO:0051338~regulation of transferase activity	0.047664
MF(level4)	GO:0008484~sulfuric ester hydrolase activity	6.83E-05
	GO:0005125~cytokine activity	2.07E-04
	GO:0005539~glycosaminoglycan binding	5.99E-04
	GO:0048029~monosaccharide binding	0.001921
	GO:0016758~transferase activity, transferring hexosyl groups	0.00566
	GO:0016706~oxidoreductase activity, acting on paired donors, with incorporation or reduction of molecular oxygen, 2-oxoglutarate as one donor, and incorporation of one atom each of oxygen into both donors	0.007786
	GO:0004091~carboxylesterase activity	0.011557
	GO:0048407~platelet-derived growth factor binding	0.011655
	GO:0048185~activin binding	0.011655
	GO:0001664~G-protein-coupled receptor binding	0.011865
	GO:0008329~pattern recognition receptor activity	0.02324
	GO:0008194~UDP-glycosyltransferase activity	0.029082
	GO:0016702~oxidoreductase activity, acting on single donors with incorporation of molecular oxygen, incorporation of two atoms of oxygen	0.029348
	GO:0042803~protein homodimerization activity	0.030521
	GO:0004866~endopeptidase inhibitor activity	0.032084
KEGG Pathway	hsa04510:Focal adhesion	2.80E-05
	hsa04512:ECM-receptor interaction	3.86E-05
	hsa04060:Cytokine-cytokine receptor interaction	7.10E-04
	hsa05412:Arrhythmogenic right ventricular cardiomyopathy (ARVC)	0.003228
	hsa00533:Keratan sulfate biosynthesis	0.003589
	hsa05410:Hypertrophic cardiomyopathy (HCM)	0.007245
	hsa04142:Lysosome	0.010139
	hsa05414:Dilated cardiomyopathy	0.012446
	hsa04810:Regulation of actin cytoskeleton	0.014848
	hsa04062:Chemokine signaling pathway	0.01747
	hsa04360:Axon guidance	0.020822
	hsa00051:Fructose and mannose metabolism	0.022076
	hsa04620:Toll-like receptor signaling pathway	0.022766
	hsa04621:NOD-like receptor signaling pathway	0.028368
	hsa00052:Galactose metabolism	0.03409
	hsa05120:Epithelial cell signaling in Helicobacter pylori infection	0.04394

hsa04916:Melanogenesis	0.047923
